# A dolphin-inspired compact sonar for underwater acoustic imaging

**DOI:** 10.1038/s44172-022-00010-x

**Published:** 2022-06-08

**Authors:** Hari Vishnu, Matthias Hoffmann-Kuhnt, Mandar Chitre, Abel Ho, Eszter Matrai

**Affiliations:** 1 Acoustic Research Laboratory, Tropical Marine Science Institute, National University of Singapore, 18 Kent Ridge Road, Singapore, 119227 Singapore; 2 Ocean Park Hong Kong, 180 Wong Chuk Hang Road, Aberdeen, Hong Kong SAR China

**Keywords:** Ocean sciences, Electrical and electronic engineering

## Abstract

Underwater imaging sonars are widely used for oceanic exploration but are bulky and expensive for some applications. The sonar system of dolphins, which uses sound pulses called clicks to investigate their environment, offers superior shape discrimination capability compared to human-derived imaging sonars of similar size and frequency. In order to gain better understanding of dolphin sonar imaging, we train a dolphin to acoustically interrogate certain objects and match them visually. We record the echoes the dolphin receives and are able to extract object shape information from these recordings. We find that infusing prior information into the processing, specifically the sparsity of the shapes, yields a clearer interpretation of the echoes than conventional signal processing. We subsequently develop a biomimetic sonar system that combines sparsity-aware signal processing with high-frequency broadband click signals similar to that of dolphins, emitted by an array of transmitters. Our findings offer insights and tools towards compact higher resolution sonar imaging technologies.

## Abstract (web summary)

Hari Vishnu and colleagues use signal processing that exploits sparsity information to visualise dolphin echolocation signals during object interrogation. The approach is then combined with transmission of dolphin-like click signals towards a compact acoustic imaging tool.

## Introduction

Underwater imaging sonars are an essential technology for oceanic exploration and have been in use for many decades in several applications. Biomimetic sonars that are inspired from marine mammals such as dolphins are an emerging development in this field^1^. The biological sonar of dolphins surpasses any current man-made imaging sonars of similar size and frequency^2–4^. Dolphins can use their biosonar to identify objects varying in size, shape, and material^5^. Behavioural studies demonstrate that dolphins can sense objects both visually and echoically, and transfer information across these sensory modes^6,7^. This behaviour is demonstrated by echoic-to-visual (EV) cross-modal matching-to-sample (MTS) experiments, in which a dolphin uses echolocation to inspect a sample, and identify the match from amongst alternative objects through its visual sense^8^.

**Fig. 1:**
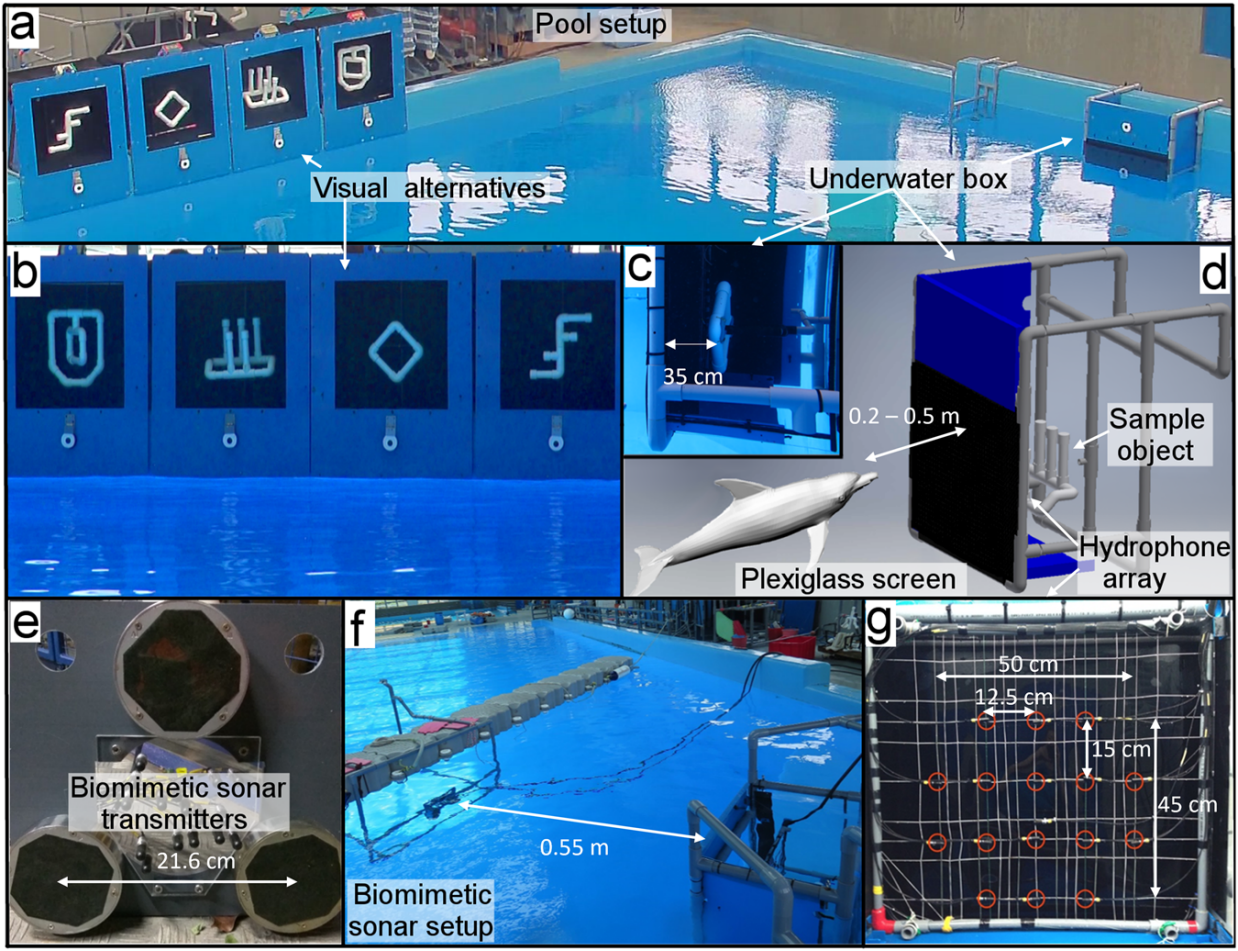
Set-up used for echoic-to-visual matching-to-sample and biomimetic-sonar trials. **a** Pool set-up. **b** Box housing the visual alternatives. **c** Underwater box containing the sample object, where the dolphin performs the echoic interrogation. **d** Schematic of the underwater set-up used for the trials indicating the sample box, object, Plexiglas screen, hydrophone array and approximate location of dolphin (or biomimetic transmitters). **e** Biomimetic transmission system with three co-located transmitters. **f** Set-up used for biomimetic-sonar trials with underwater transmitters facing the sample object. **g** An array of 16 hydrophones is placed behind the Plexiglas screen covering the sample object, recording the acoustic information during the trials.

Obtaining a deeper understanding of how dolphins process echolocation information is challenging. The dolphin brain and sonar are complex systems, which makes it hard to examine their individual aspects like shape-recognition, without isolating others such as behavioural biases. Moreover, the instrumentation required to record or transmit dolphin-like signals with high frequency and bandwidth has only been slowly evolving over the past decades^4^. In order to better understand the shape-recognition capabilities of dolphin biosonar with an aim to replicate it in a biomimetic system, we conduct EV-MTS experiments in a pool^8–10^ (Fig. 1a). In these experiments, the dolphin is able to perform certain target-discrimination tasks. This allows us to better observe the capabilities of dolphin echolocation using high-frequency recording equipment. Furthermore, we develop a biomimetic-sonar system that mimics the dolphin’s biosonar by using (1) a broadband dolphin-like transmit signal, (2) emitted by high-frequency transmitters placed at different locations, and (3) multiple repeated clicks. We use this to insonify the same objects used in the EV-MTS trials and analyse the recordings. The aim is to determine what sonar performance we can obtain and what processing may be required to differentiate targets as effectively as the dolphin. From a practical viewpoint, this helps evaluate techniques that may help enhance the performance of man-made sonar.

While dolphins are capable of shape recognition, it is unclear how they perform this task well given their limited sensory aperture. In some cases, dolphins may also have to face noisy scenarios where the transmit energy they can expend in each click may be inadequate. Their use of repeated interrogation clicks during transmission^11^ may help them overcome noise-induced false alarms because target returns are often consistent while noise is not^12^. Furthermore, prior research shows that dolphins use beam-steering in transmissions during target recognition^8,13^. This combined with the dolphin’s movement may ensure that different aspects of the targets are adequately insonified via a multi-look evaluation, which is important to overcome masking effects that may hide features in some cases^12,14^.

For the reception, one modality possibly used in odontecetes is via their lower-jaws^11,15–19^. Irrespective of its details, the aperture of a dolphin’s sensor array while acoustically scanning a target is limited in the cross-sectional plane of its head, which has a diameter *D* usually less than 20 cm^20^. Considering this limited size, dolphins perform well in terms of fine angular resolution, viz. the ability to distinguish small details or features located close to each other. We try to quantify this in the context of conventional narrowband sonar, in the following. Dolphins are known to transmit clicks with different spectral content^21^. For typical bottlenose dolphin clicks with most energy within 110–130 kHz^11^ (Supplementary Fig. 2) which correspond to type-E clicks as classified by Houser et al.^21^, a nominal click frequency is around 120 kHz. When acoustically imaging using a head-sized receiver and a signal of wavelength *λ* using narrowband sonar, the angular resolution is ~*λ*/*D* radians^22^. Based on this, we would expect its lower limit on an angular resolution to be around 3.6°. However, dolphin experiments^23^ show they can achieve a angular resolution as fine as 1°.

This performance looks impressive also in light of the number of sensors that a narrowband sonar receiver would need to achieve this. Even if we consider a receiver aperture spanning a dolphin’s head size, a large number of sensors covering this region would be required to adequately perform acoustic sensing. This is because narrowband processing requires that neighbouring sensors cannot be separated by more than half a wavelength of spacing. Exceeding this limit leads to spatial aliasing—duplication of targets in the sonar’s output visualisation in the form of repeated images known as grating lobes^22^. If we were to use a two-dimensional circular head-sized sensor array with area *πD*^2^/4, the number of sensors required to fully populate it and avoid aliasing would be $> \frac{\pi {D}^{2}}{4{\left(\lambda /2\right)}^{2}}$, i.e. at least 773. Using current man-made technology, it is impractical to design or fabricate arrays with such a large number of sensors packed within a small region, let alone process such a sizeable amount of data. Thus, man-made sensor arrays operating at these frequencies and aperture would have to be sparse, i.e. with fewer sensors than necessary to avoid spatial aliasing.

One prominent advantage of dolphin biosonar that allows it to outperform narrowband sonar is its use of broadband signals^12,21,24^. A broadband frequency-domain sonar processing approach using such signals can reduce the effect of grating lobes to some degree. Such processing has been used for building bio-inspired sonars^14,25^, including ones that use dolphin-like signals^24,26–30^, and the performance advantages of using these signals have been highlighted^12,31,32^. Biomimetic transmission systems that are able to produce narrow directed beams have been designed^18,33–37^, and bio-inspired receptors that mimic those of porpoises have been developed^38^, with potential applications to miniaturised sonar systems. However, a compact sonar still faces the limitation imposed on the reception due to sensory aperture. Replicating the transmission system using a broadband signal combined with multi-look transmitters alone does not solve the problem without infusing additional information, as we demonstrate later on. Given these challenges, the performance demonstrated by the dolphin sonar gives us much to aim for. Some studies tested whether the dolphins’ movement during echolocation is key to their superior performance via an approach similar to synthetic-aperture scanning, but found that movement is not essential to the performance as they can use beam-steering and shaping^8,9^.

While dolphins could use simple acoustic features such as target strength of the echoes to perform echoic–echoic matching of objects^5,39^, these are not necessarily helpful for EV-MTS tasks. In many cases, dolphins have been shown to match object shapes across visual and acoustic senses even the first time they are presented with an object^7,40^. Some hypotheses have been put forward that echolocation yields some mental pictorial representation of the object^41^. In any case, it is obvious that the dolphin’s echoes during EV-MTS trials contain enough information to reconstruct the shapes that it acoustically interrogates, or at least features that allow target discrimination. There are no visualisations of these in terms of target shapes in an interpretable form using multi-sensor array recordings yet, though previous works have used such arrays^13,42–44^ for objectives like testing the transmit-beam focusing hypothesis.

Here, we investigate what sonar processing is required to reconstruct the object shapes that the dolphin acoustically interrogates, using the echoes received by the dolphin during the EV-MTS trials. Subsequently, we replicate this processing on a biomimetic sonar. We elucidate that infusing information on target sparsity into the processing allows us to acoustically visualise the object’s features better than conventional techniques. We then examine the discrimination of these targets using a quantitative metric. We show that the improved sparsity-aware processing enables target discrimination using a compact sonar set-up, thus taking us a step towards closing the performance gap with dolphins who are able to discriminate the same targets. This technology can be used for improved acoustic imaging, especially in underwater environments where sound is an ideal sensing medium— sound waves travel longer distances in sea-water than electromagnetic waves^1^. The sonar’s compactness can make it easier to mount on underwater robots used for ocean exploration.

## Experimental design

The subject of this study is a 10-year-old male Indo-Pacific bottlenose dolphin (*Tursiops aduncus*) named Ginsan, housed at the Marine Mammal Breeding and Research Centre at Ocean Park, Hong Kong, who has training and experience in EV-MTS tasks and has been the subject of previous similar research^8–10^. Research sessions consist of either EV-MTS trials (Fig. 1), or biomimetic-sonar trials which replicate the acoustic part of the former for a performance comparison. The EV-MTS trials’ objectives are to examine the dolphin’s capability to match objects across sensory modalities (acoustic to visual), with the goal of creating a biomimetic system to achieve good discrimination performance under similar settings.

**Fig. 2:**
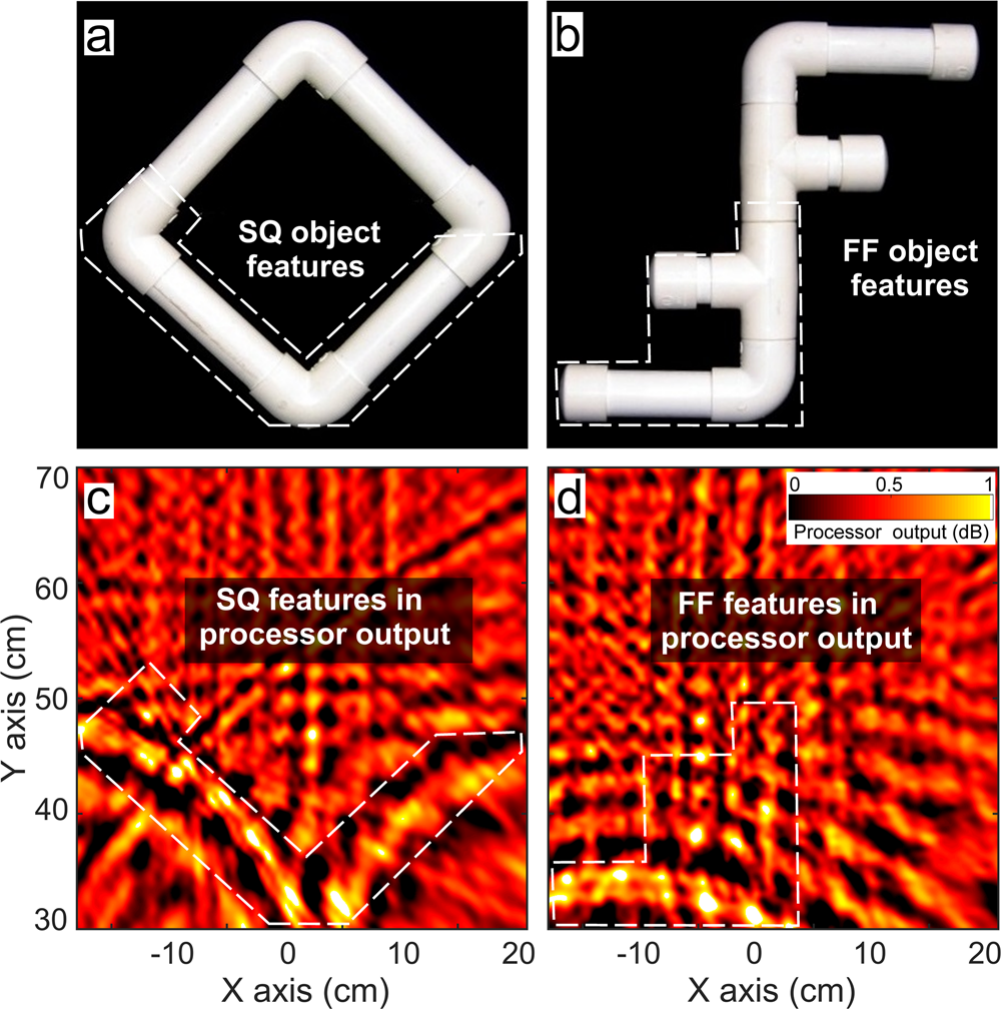
Bartlett processor visualisations with dolphin-echolocation data. **a**, **b** SQ and FF objects used in the echoic-to-visual matching-to-sample experiments, respectively. Bartlett visualisations using (**c**), acoustic dataset #1 for SQ and (**d**) acoustic dataset #2 for FF sample objects, highlighting shape features that can be matched to the sample's shape (white dashed lines). The discrimination coefficients of these visualisations are 0.046 dB and 0.005 dB (Table 1).

During the EV-MTS trials (‘Methods’), Ginsan has to match different sample objects to one of several alternatives visually^8^. Sample and alternative stimuli are presented to Ginsan’s echoic and visual senses respectively (Fig. 1a–d and Supplementary Movies 1 and 2). This ensures that the dolphin extracts information on the object’s shape or its features from its click echoes, to perform the task. In order to present stimuli to his echoic sense only, an underwater anechoic box is developed (Fig. 1d). Several sample shapes were used in the trials, of which four are considered in this study, named SQ (square, Fig. 2a), FF (double F, Fig. 2b), OC and EL. SQ and FF are equated for reflective surface area, thus ensuring their overall reflectivity is comparable.

The biomimetic-sonar trials are set up in a similar way as the EV-MTS trials, mimicking the scenario during the dolphin’s echoic interrogation, with the biomimetic-transmitter system replacing the dolphin. During these, the system is mounted in the pool in front of the object. Its size is comparable to a dolphin head, and it consists of three co-located transmitters whose main beams are pointed at the object (Fig. 1e). The transmitted signals are broadband with a centre frequency of 120 kHz similar to the type-E dolphin clicks^21^ recorded by us (Supplementary Fig. 2). Each transmitter emits a click-train inspired by the repeated interrogation approach used by dolphins^12^. Three transmitters are used to ensure diversity in the angle at which the object is insonified. Using each transmitter, we obtain different aspects of the object during imaging, reducing the chances of missing out object features due to shadowing (further discussion in ‘Methods’). This is inspired by the dolphin’s beam-steering capability^8,13^, which allows it to target different parts of the objects during echolocation. The biomimetic-sonar captures three such different aspects, so its coverage is limited compared to a dolphin which is free to use more beam directions during interrogation. Moreover, the fixed set-up cannot fully emulate any advantage the dolphin may obtain due to its movement, although this is partly captured by the three different locations of the transmitters. Previous work suggests that movement may not be essential to the dolphin’s performance^8,9^. For both types of sessions, a planar array of 16 sensors is placed in the sample box recording the acoustic information (Fig. 1d, g). Its width and height are roughly double the diameter of a bottlenose dolphin’s head.

## Data preprocessing and modelling

**Fig. 3:**
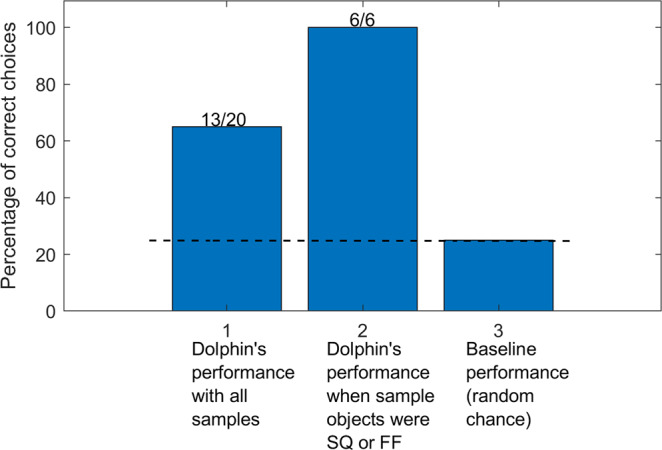
Dolphin’s performance in 4-alternative echoic-to-visual matching-to-sample trials. Ginsan was correct on 13 of his choices out of 20 trials where several different sample objects were used in the study. He got 6 choices right in all 6 trials where the sample was either SQ or FF, two of the shapes considered in the current study.

For the dolphin-echolocation trials, we use four acoustic datasets recorded in September 2014 numbered #1 to #4, which contain recordings of Ginsan’s transmissions and their echoes with a high signal-to-noise ratio (SNR). During the sessions in September 2014, Ginsan is able to find the correct alternative in 13 out of 20, i.e. 65%, of the 4-alternative trials where several different sample objects were used in the study^8,9^ (Fig. 3). As compared to a baseline random-chance score of 25%, this is significantly higher, statistically (*P* = 1.837 × 10^−4^, *n* = 20 independent experiments), showing that Ginsan performs better than random-guessing using the cues from acoustic interrogation. Specifically, Ginsan is correct in all six of the tasks where the samples are SQ or FF, i.e. 100%, which is also significantly higher than random chance (*P* = 2.441 × 10^−4^, *n* = 6 independent experiments). From the biomimetic-sonar trials, we use four datasets numbered #5 to #8 containing biomimetic transmissions and echoes.

We preprocess the data to extract the listening periods for all clicks employed for the interrogation. The main processing step involves applying a source-localisation technique based on matched-field array processing^22,45^. Based on the available data, the processor visualises the region insonified by the dolphin or biomimetic transmitters during the experiment. To do this, we first require a forward model of the echoes corresponding to each transmitted click based on the physics of the set-up, which we develop. All these components are described in ‘Methods’.

## Results

### Bartlett processing

Standard array-processing-based visualisation techniques like Bartlett have been in use for a long time^22,45^. To tap into the information across the available bandwidth in the echoes, we use broadband Bartlett processing which averages the output for different frequency bands (see ‘Methods’). This is able to exhibit some shape features of the sample in its visualisation (Fig. 2c, d). However, these visualisations are noisy due to the effect of grating lobes, which arise in the output at each frequency band when conventional processing is applied on data from an array of sensors separated by a distance greater than *λ*/2^22^. Our array’s sensors are separated by at least 12.5 cm, which exceeds the half-wavelength limit for the frequencies considered. Broadband Bartlett processing smears out these grating lobes to some degree via the naive procedure of averaging across frequency bands, but does not suppress them entirely.

We now pose the question—are there enough features in the processor output to distinguish the sample in the box from another alternative? To answer this, we superpose the shapes of the candidate alternatives onto the outputs, and evaluate which of the superpositions show more overlap. We compute the amount of overlap as the correlations of outputs against shapes of the sample and alternative object, based on matched filtering (‘Methods’). A metric called discrimination coefficient *R*, represents how much more the output is correlated with the sample rather than the alternative. This is a measure of how well the processor output enables us to choose the correct sample versus an alternative. We specifically examine discrimination between the objects SQ and FF (Fig. 2a, b) since the dolphin exhibited more proficiency in distinguishing these in the EV-MTS trials. If the sample for a trial is SQ, the alternative compared against is FF, and vice versa. Datasets #1 to #6, which have either SQ or FF as the sample, are considered for testing SQ-FF discrimination. The *R* values computed for Bartlett outputs (denoted with _B_) for datasets #1 to #6 are tabulated in Table 1 (data available in ref. ^46^).

**Table 1:** Discrimination coefficient *R* computed for SA and Bartlett processor outputs from different datasets.

Dataset number	Sample object	*R*_B_ (dB)	*R*_SA_ (dB)
1	SQ	0.046	3.97
2	FF	0.0053	1.44
3	SQ	0.0057	1.26
4	FF	0.0149	1.85
5	SQ	0.21	3.6
6	FF	0.020	1.92

A larger value of *R* indicates that the processor output contains more features showing evidence of the correct sample’s presence against the alternative.

The *R*_B_ values for the dolphin-echolocation data (#1 to #4) are close to zero. This indicates that the Bartlett outputs computed on the same acoustic data available to Ginsan during the task are not clear enough to facilitate target discrimination. We recollect that the dolphin completed the EV-MTS task successfully in 100% of the trials. One reason is that the dolphin’s sonar system inherently possesses more information such as the transmit time and signal, which are unknown to us, because so far we have only been ‘listening in’ to its interrogation. This leaves our processors at a disadvantage when processing the dolphin-echolocation data, though the exercise reaffirms to us that the acoustic information can be visualised, and that we should aim for better performance. In order to gauge our processors’ performance without this disadvantage, we test discrimination of SQ and FF with our biomimetic sonar, which makes the transmit time and signal available to us.

**Fig. 4:**
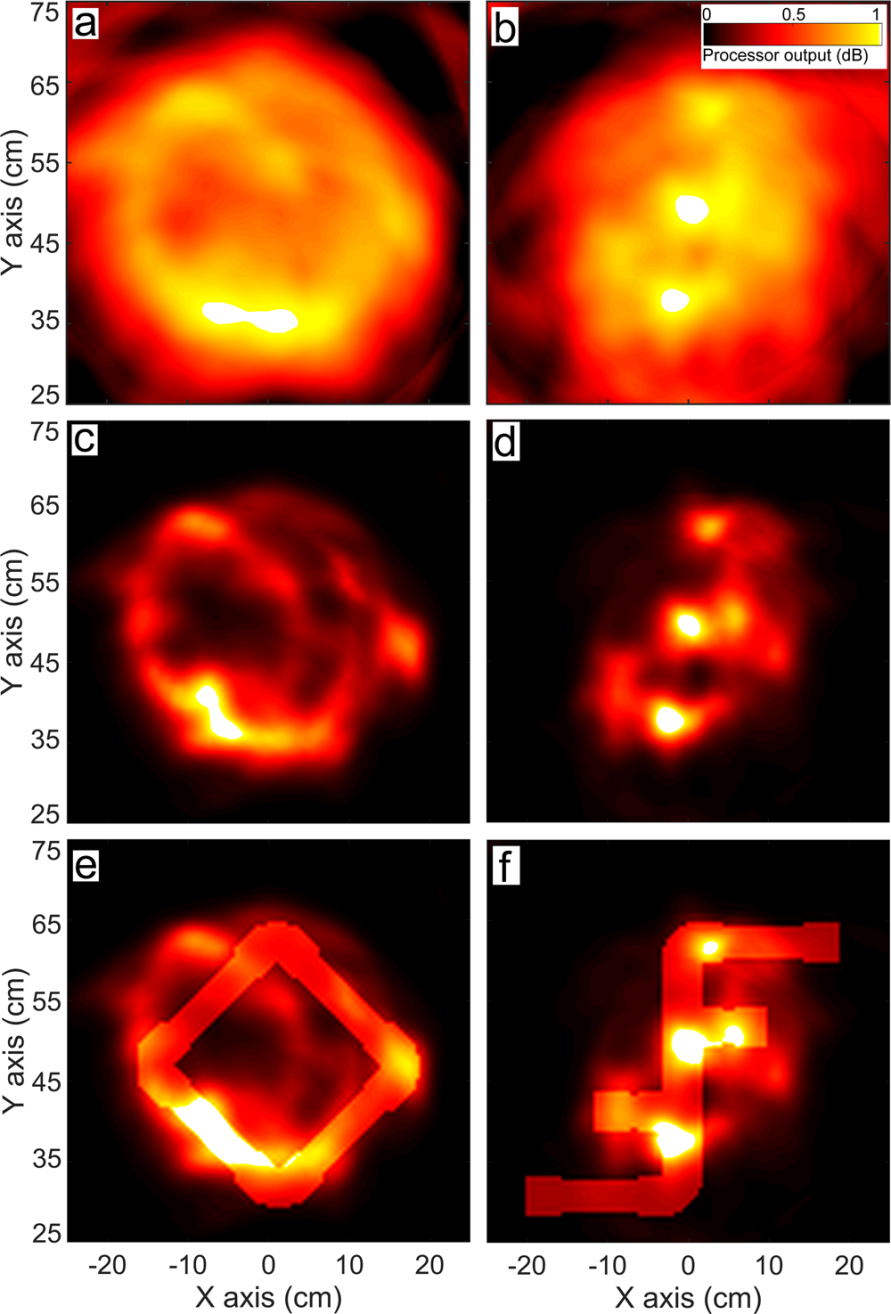
Comparison of biomimetic-sonar data visualisations using sparsity-aware (SA) and Bartlett processing. **a**, **b** Bartlett visualisations with datasets #5 and #6 respectively. Discrimination coefficients of these are 0.21 dB and 0.02 dB. **c**, **d** SA visualisations for datasets #5 and #6 respectively. **e, f** SA visualisations for datasets #5 and #6 respectively shown with shading masks in the shape of the samples highlighting the matching features. The discrimination coefficients of these are 3.6 dB and 1.92 dB, respectively.

When visualising the biomimetic-sonar echoes, Bartlett processing (Fig. 4a, b) performs better than it did with the dolphin-echolocation data (Fig. 2a, b), due to the incorporation of knowledge of the transmit time, signal, and source location. However, the outlines in the Bartlett outputs are still blurry and the discrimination coefficients are not large, indicating some features are observable but not clear enough to facilitate confident discrimination of the objects. The processor’s limited discrimination performance does not seem to add up given that the dolphin was able to pick the correct alternative in all the EV-MTS trials where the sample is SQ or FF. This inspires us to move beyond conventional processors to obtain better visualisations that facilitate clearer target discrimination using additional prior information. Humans, too, are known to use prior information for sensing with limited data^47–50^.

### Sparsity-aware broadband processing

To bridge the performance gap noted above, we design a smarter processor that uses information missing in the conventional approach discussed so far. The first piece of information that is not effectively used in the Bartlett approach is the prior knowledge on the sparsity of the object. The samples explored in this study occupy only a small fraction of the space within the interrogated box. Moreover, each click results in well-defined acoustic returns from only some portions of the objects. We infuse this information into our processing by tuning it to search for sparse solutions, so that it paints the target with only a small number of voxels.

The second piece of information that is not efficiently used in Bartlett's processing of the dolphin-echolocation data is the broadband nature of the clicks (Supplementary Fig. 2). In the Bartlett approach, recall that we are limited to computing an output for each frequency component separately. Subsequently, we average the output components across frequencies, leaving the final image noisy (Fig. 2c, d). This does not effectively utilise the information across multiple frequencies. For example, voxels that the processor evaluates as being occupied by the object at some frequency bands, may not be evaluated as being occupied at other bands, and in the final stage, an average of these separate inconsistent evaluations at different frequencies is visualised. Better suppression of noise and grating lobes can be gained if the multi-frequency information is utilised during the processing itself, but we are forfeiting this if we combine this information at the output stage after processing using the conventional method.

How can we use broadband information better than conventional processors? To answer this, note that if a recording contains a broadband echo, it manifests as a simultaneous energy increase across a large spectrum of frequencies (Supplementary Fig. 2). This information is particularly useful when a broadband signal is considered because the processor can check for consistency across a larger number of frequency bands. We use this information by designing an approach that seeks solutions where there is consistency in the energy level across frequencies corresponding to detected echoes, during the processing itself. In doing so, we better exploit the broadband advantage offered by the dolphin’s transmit signal.

To improve our visualisations and incorporate these two pieces of information, we develop a sparsity-aware (SA) processor based on the compressed sensing philosophy (‘Methods’)^51,52^. Compressed sensing-based approaches work well in reconstructing signals from sparsely sampled data^52^. The SA processor ensures sparsity information is used by minimising a cost function based on a *p*-norm (with *p* equal or close to 1) to obtain its output^53^. Consistency information is used by ensuring the visualisation is consistent across frequency bands considered (detailed explanation in ‘Methods’).

**Fig. 5:**
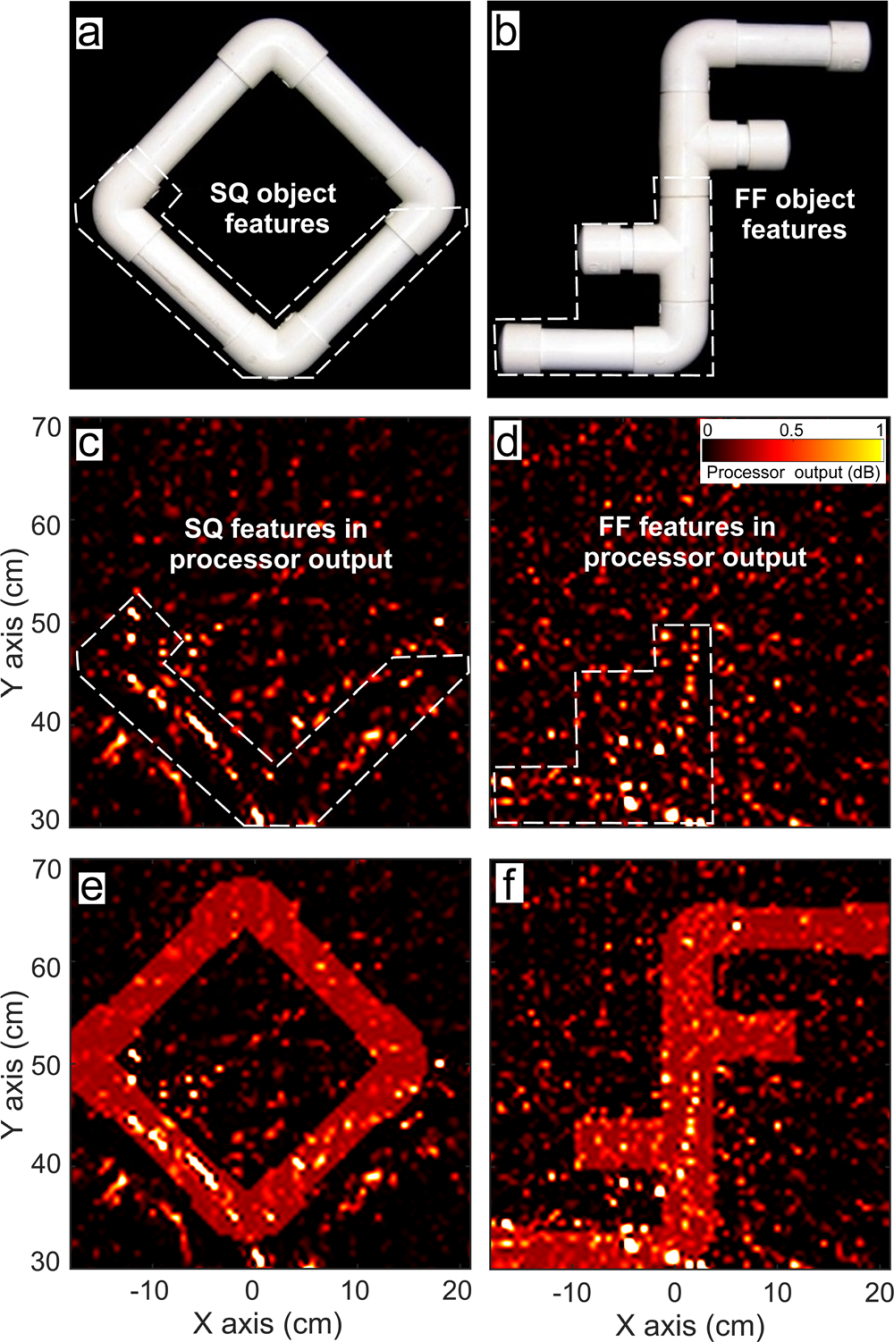
Sparsity-aware (SA) processor visualisations with dolphin-echolocation data, showing improvement over Bartlett processing (Fig. 2). **a**, **b** SQ and FF objects used in the echoic-to-visual matching-to-sample experiments. **c**, **d** SA processor visualisations using datasets #1 and #2, highlighting shape features that can be matched to the sample's shape (white dashed lines). **e**, **f** SA processor visualisations using datasets #1 and #2 with superposed shading masks in the shape of the respective sample, highlighting matching features. These visualisations yield discrimination coefficients of 3.97 dB and 1.44 dB, respectively.

**Fig. 6:**
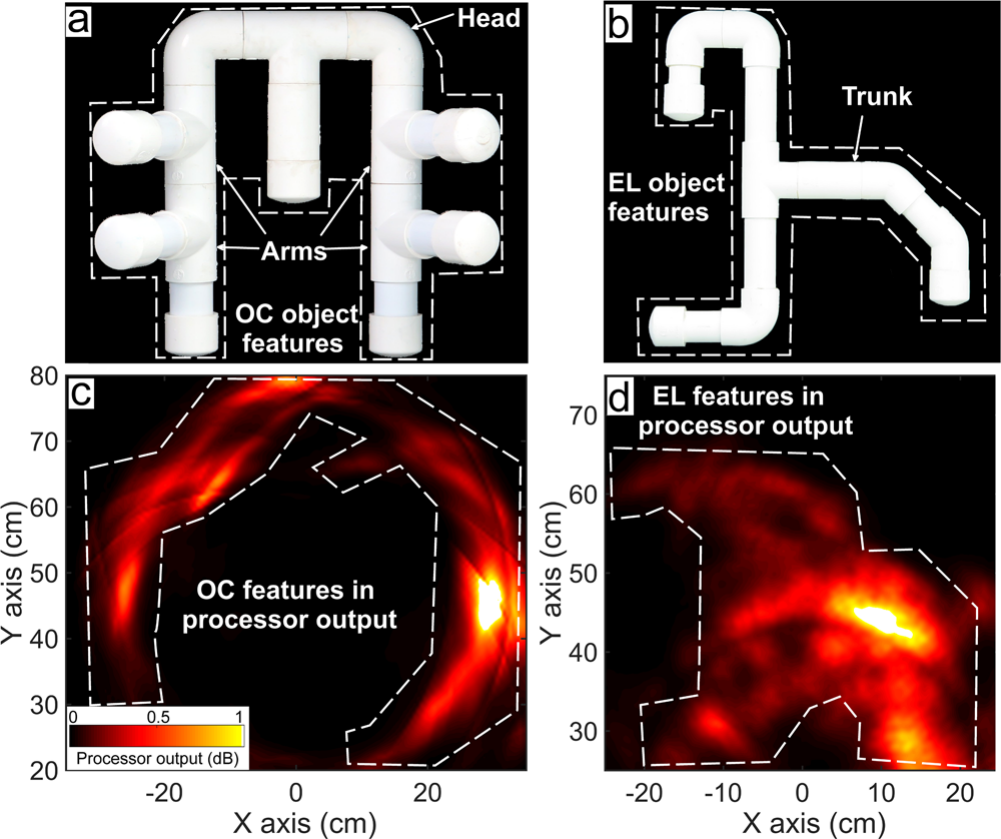
Sparsity-aware processor visualisations with objects OC (octopus) and EL (elephant). **a**, **b** The sample objects OC and EL. **c** Output from dataset #7 where OC is the sample. **d** Output from processing the first transmitter's click in dataset #8 where EL is the sample. The visualisation using all three transmitter clicks is similar but with the horizontal bar on the right more prominently seen (see Supplementary Fig. 4).

We generate SA processor outputs for the dolphin-echolocation datasets (Fig. 5 for #1 and #2) and biomimetic-sonar (Fig. 4, c and d for #5 and #6). We also tabulate the discrimination coefficients *R*_SA_ (for SA processing) with datasets #1 to #6, in Table 1 (data available in ref. ^46^). A comparison of Figs. 5 and 2, and the panels of Fig. 4 demonstrates that SA processing yields clearer outputs than Bartlett - the grating lobe levels and fuzziness are suppressed, and the outputs show discernible shape features matching the sample objects. Additional results using the SA processor with dolphin-echolocation datasets #3 and #4 (Supplementary Fig. 3) and biomimetic-sonar datasets #7 and #8 (Fig. 6) further elucidate the processor’s capability to capture some features of the sample in its visualisation. Figure 6 shows the outputs for the objects OC (Fig. 6a) and EL (Fig. 6b). Although some features are missing or distorted in the visualisations, there are enough to distinguish the objects.

For all datasets considered, the large *R*_SA_ values which are greater than the corresponding *R*_B_ values (Table 1) further illustrate that SA processing does better than conventional processing and yields enough features to distinguish the sample object from an alternative. The output components that contribute to large *R*_SA_ values are shown by superposing the shape masks in Figs. 4e, f and 5e, f.

**Fig. 7:**
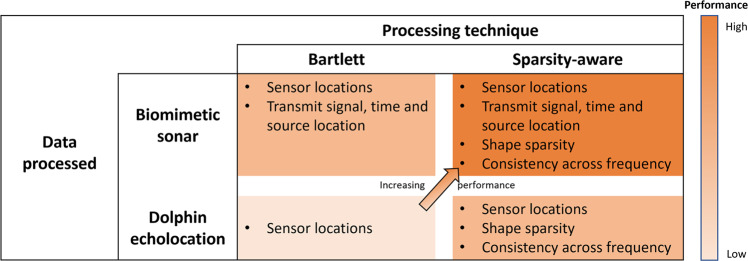
Information utilised in the different processing techniques and types of experimental data used in the study. The Bartlett processor applied to the dolphin-echolocation data uses the least amount of information, whereas the sparsity-aware processor applied to the biomimetic-sonar data uses the most information. Consequently, the performance of the SA processor with biomimetic-sonar data is the best amongst the approaches considered.

A table comparing the information used by different approaches is shown in Fig. 7. The Bartlett processor applied to the dolphin-echolocation data uses the least amount of information, whereas the SA processor applied to the biomimetic-sonar data uses the most information. Consequently, the performance of the SA processor with biomimetic-sonar data (Fig. 4e, f) is the best. Furthermore, we demonstrate the benefits of repeated interrogation in the SA processor outputs in Supplementary Fig. 5. The use of additional clicks yields clarity improvement in the processor outputs due to better noise suppression.

## Discussion

Dolphins’ superior acoustic imaging performance compared to man-made sonars clearly indicates a sophisticated processing system well-evolved for the task. We conduct the EV-MTS trials to examine their shape-discrimination capability, by guaranteeing that Ginsan discerns the sample shape from the acoustic data to perform his task. This allows us to gauge our biomimetic sonar under similar conditions and gives us a performance goal to shoot for. Ginsan’s good performance in the EV-MTS trials indicates there is enough information in the echoes received during acoustic interrogation to discriminate the shapes from alternatives. We visualise the acoustic information by generating a spatial representation from the received echoes using matched-field processing. To the best of our knowledge, this is amongst the first attempts that successfully visualise the acoustic information in dolphin-sonar echoes with array recordings. Since the visualisations are formed from the same information available to Ginsan, they allow us a fair evaluation against dolphin biosonar using our own processing.

Our biologically inspired sonar system uses high-frequency broadband transmitters emitting dolphin-like signals, repeated interrogation with multiple clicks, and different transmitter locations to get multiple aspects of the target, similar to the advantages a dolphin-biosonar system enjoys. If conventional processing is used, the visualisation clarity is limited because the reception system’s compactness and sparse sampling leads to grating lobes in the output, and the target-discrimination performance possible with this does not look as convincingly good as the dolphin’s. This demonstrates that with the given hardware and physical set-up, the target-discrimination performance we can achieve is limited if additional information is not used. We surpass this limit using SA processing, producing clearer outputs where grating lobes are suppressed using the information on sparsity of the targets, and information on the cross-frequency consistency of the signals. This improved processing enables better shape discrimination using data from both the dolphin and biomimetic-sonar. This elucidates how incorporating additional prior information such as sparsity, and better exploiting the broadband nature of the signal, can help us improve upon the conventional processing that is widely used in man-made sonars today.

The SA processing method is powerful enough to reconstruct identifiable shape features using just three clicks (one per transmitter) allowing it to be operationally fast. Furthermore, it uses only 15 sensors which are undersampling the space and span a size on the order of the dolphin’s head size, thus beating the size-performance trade-off faced by current-day sonars. If additional clarity is needed in noise-limited environments, the sonar’s repeated interrogation feature can help it overcome the noise by using multiple clicks until the image is clear enough.

Overall, this compact biomimetic sonar is able to visualise shapes and facilitate target-discrimination underwater. This system could be of use in underwater sensing, or feature identification for navigation. Its compact size makes it suitable for mounting on small underwater vehicles, paving the way for next-generation oceanic exploration.

## Methods

### Methodology for EV-MTS trials

Ea ch trial is conducted as follows^8^. At the start of each trial, a sample object is placed inside the box 20 cm away from the screen in the dolphin’s absence. Alternative objects are presented in air in display boxes (Fig. 1b), thus ensuring they are accessible to the dolphin’s visual sense only, as he cannot effectively echolocate in air^7^. During the trial, an assistant switches on a bubble curtain in front of the object to block any echolocation before the dolphin is positioned in front of the box. When the dolphin is positioned to start his interrogation, the bubble screen is switched off via a sensor which is synchronised with the acoustic recordings. The trainer sends Ginsan into the experiment pool. The dolphin enters the pool and echoically interrogates the sample object in the box underwater, while his transmissions are recorded. Once Ginsan has enough information, he swims over to the other side of the pool where the alternatives are placed. Via this set-up, Ginsan is allowed to freely control the duration and position of his acoustic interrogation, and also to move around during this period. On a cue by the experimenter, the blinds covering the objects are pulled up revealing all visual alternatives simultaneously. The dolphin inspects the alternatives and presses the response paddle underneath his chosen object. An external observer announces if the dolphin made a correct choice and the trainer reinforces the animals with a whistle and a fish if the choice is correct. After a brief interval during which the sample stimuli are changed, the next trial is started. During this, the dolphin stays in an adjacent pool, thus preventing him from seeing the objects placed inside the box or prematurely echolocating on the next sample. Similarly, to ensure blind and unbiased trials, neither does the person operating the sample box know what alternatives had been placed in the alternative boxes, nor does the person operating the alternative display boxes have any knowledge of the sample object. The trainer of the dolphin also stays at the entrance of the pool and does not interact with the dolphin until the trial is over. Moreover, the box setups used ensure that no unintentional cueing of the dolphin is possible. Steps are also taken to randomise the objects across the trials. During the 20 trials, the alternatives are all placed in different orders using a prepared pseudorandom configuration, and each of the four locations have the correct choice an equal number of times. The samples presented to the dolphin are also selected using a pseudorandom configuration, which ensure that the same sample does not repeat consecutively, in order to reduce recency bias on part of the dolphin. The sample configurations during sessions also ensure that all four objects of the study are repeated an equal number of times, thus ensuring it was a balanced study. This research was fully approved by the Institutional Animal Care and Use Committee of both the National University of Singapore and Ocean Park Hong Kong, and all experiments were performed in accordance with relevant guidelines and regulations.

### Construction of underwater sample box used in trials

The underwater anechoic box is developed from polyvinyl chloride (PVC) schedule-80 pipe and fittings to house the sample object, with an opaque Plexiglas screen in the front that is transparent to sound (Fig. 1d)^8^. Attached to the side panels are 6-mm-thick neoprene sheets that block echolocations from entering from the side. Thus, acoustic sensing is the only way for Ginsan to perceive the sample. The objects are made from 32 mm diameter white schedule-40 PVC pipe and fittings filled with air, which increases their acoustic reflectivity underwater. The objects are suspended in the box using a PVC holder that can be lowered and attached to the rear side of the box, ensuring that they are always placed in the same location during the trials.

### Biomimetic-sonar hardware

The transmitter is a custom-made split beam transducer^54^ with a nearly flat frequency response within 105–140  kHz. The 10-dB beam-width at 120 kHz is roughly 20°, which is wide enough to ensure that all three transmitters beams cover the insonified sample object at the range considered, in line with recommendations in previous biosonar work^12^. The total sonar device size is within a 36 cm diameter circle excluding the mechanical parts used for mounting, which is comparable to the size of a dolphin head. During the trials, the sonar is mounted at a position similar to the dolphin’s location during the EV-MTS trial. For transmissions, a pre-recorded short-duration broadband click signal which has good autocorrelation, is fed to the transmitter at 2.5 MHz sampling rate to obtain good time resolution on the transmitted signal. The click repetition rate is set at 80 Hz, based on the observed rates used by dolphins for object interrogation at the target ranges considered in the EV-MTS trials. Each transmitter transmits for 5 s each.

### Recording hydrophone array

For both the EV-MTS and biomimetic-sonar sessions, an array made of 16 Reson TC4013 miniature reference hydrophones^55^ is placed in the sample box 3 cm away from the Plexiglas screen recording the acoustic information (Fig. 1d, g). The hydrophones’ frequency response is nearly flat within 5–140 kHz, and has a sensitivity of -211 dB ± 3 dB re 1 V/*μ*Pa. The array was built by constructing a frame (87 × 78 cm) from 16 mm schedule-80 PVC pipe and fittings, and its size is comparable to that of the dolphin’s head. The signals from the array are acquired after preamplification via two National Instruments data acquisition systems consisting of a PXIe-1062Q^56^ 8-Slot 3U PXI Chassis, a PXIe-8108 Core 2 Dual 2.53 GHz Controller and two National Instruments PXI-6133 32 MS Memory Series Multifunction data acquisition systems. Data are acquired with a custom-written MATLAB software at 500 kSamples per second per channel, which covers the frequency range containing most energy within the clicks^11^.

### Selection and preprocessing of acoustic data to extract echoes

For dolphin-echolocation data analysis, we use four acoustic datasets recorded in two sessions in September 2014 with high SNR in the 50–170-kHz band. These consist of 10-s-long time series recorded at a sampling rate of 500 kHz with 16-bit resolution. The data contain 150–500 echolocation clicks transmitted by Ginsan (Supplementary Fig. 2a). For matched-field array processing, we choose from amongst trials in which Ginsan’s response in the task is correct, since the acoustic data in these trials is more likely to contain adequate information for target discrimination. The clicks are transmitted with a duration of roughly 7.5 ms between them. The listening period in the data after each transmitted click contains echoes returning from the object (Supplementary Fig. 1b). We preprocess the data to extract the listening periods for all the clicks.

In the case of the biomimetic-sonar trials, the click transmissions are synchronised with the recordings. Hence, in this case, we select listening windows based on the expected arrival time of the echoes. Since the transmit signal is known beforehand, we matched-filter the recorded data with the transmit signal to improve the SNR of the received echoes^57^.

A fast Fourier transform is applied on the data to convert it into a frequency-domain form that we use for further processing. In the case of EV-MTS trials, the echoes are not received equally strongly at all the hydrophones. Moreover, Ginsan’s clicks vary in their energy content at different frequencies across the spectrum. Thus, the SNR of the received echoes in the data varies across both space (hydrophone locations) and frequency. For array processing, we only use the frequency bands at each sensor where the average SNR across all clicks exceeds a threshold of 0 dB. One out of the 16 sensors mounted on the Plexiglas screen is faulty, and the acoustic data from the remaining 15 is processed.

### Statistical significance testing

The statistical significance of Ginsan’s performance improvement compared to random chance (25%) is analysed with the Scipy package in the Python programming language using a one-tailed binomial test. A *P* value less than 0.01 is considered statistically significant.

### Transmit click identification and analysis in dolphin acoustic data

A spectrogram of dolphin-echolocation acoustic data from dataset #1 is shown in Supplementary Fig. 2a. The transmit clicks are broadband in nature, and the frequency content of clicks varies across time. This matches observations in the literature^21^ which note that dolphins employ clicks with differing spectral content. In this work, we do not process clicks with different spectral content differently, but rather focus on a single frequency band for processing all the clicks. To identify the transmit clicks in the data, first the data is bandpass filtered within (50, 170) kHz because the average SNR of transmit clicks across all sensors is usually highest in this band (Supplementary Fig. 2b). Using the timings for when the bubble curtain is turned off as a cue, the locations of the clicks are identified in the recordings made at one sensor with large SNR. This is done by first thresholding the acoustic data to identify large bursts of energy, and shortlisting the clicks amongst these by taking into account the minimum separation observed between clicks (at least 4 ms) (Supplementary Fig. 1b).

### Creating a noisy dataset from biomimetic-sonar data

To test the effect of noise in the data, we add synthetically generated white Gaussian noise samples^57^ denoted by the matrix **N** to the echoes extracted from the listening period in biomimetic-sonar dataset #5, denoted by the matrix **S**. The standard deviation of noise added is adjusted so as to obtain data with SNR of 20 dB. SNR is defined in decibels as $10.{\log }_{10}\frac{\parallel {{{{{{{\bf{S}}}}}}}}{\parallel }_{2,2}}{\parallel {{{{{{{\bf{N}}}}}}}}{\parallel }_{2,2}}$, where ∥.∥p,q denotes the p, q row-norm of a matrix—a q-norm across each row of the matrix followed by p-norm along the resultant column. Noise samples are generated using MATLAB.

### Forward model for acoustic data

We present a frequency-domain model for the echoes received at the hydrophone array. In the following, ${{\mathbb{C}}}^{M\times N}$ denotes the set of complex matrices of dimension *M* × *N*. Assume the region being interrogated spans a 3D *x*–*y*–*z* Cartesian space defined by the limits $x\in [{x}_{\min },{x}_{\max }]$, $y\in [{y}_{\min },{y}_{\max }]$, $z\in [{z}_{\min },{z}_{\max }]$. The axes’ origin is located at the centre of the back wall of the sample box. The *z* coordinate is considered positive in a direction pointing from the wall towards the Plexiglas screen.

Assuming the hydrophones are omni-directional, the received signal *x*(*m*, *f*, *k*) at the *m*th sensor at frequency *f* during the *k*th click’s listening period is given by

1 $$x(m,f,k) = \int\nolimits_{{z}_{\min }}^{{z}_{\max }}\int\nolimits_{{y}_{\min }}^{{y}_{\max }}\int\nolimits_{{x}_{\min }}^{{x}_{\max }}\zeta (f,k){G}_{x,y,z}{\nu }_{x,y,z}(k)\\ {\beta }_{x,y,z}(f,k){\gamma }_{x,y,z}(m,f)\,dx\,dy\,dz,$$

where *ζ*(*f*, *k*) represents the transmitted signal’s Fourier transform coefficient at *f*, *ν*_*x*,*y*,*z*_(*k*) is the directivity of the acoustic source (dolphin/biomimetic-sonar) towards the location (*x*, *y*, *z*), *β*_*x*,*y*,*z*_(*f*, *k*) is the transmission coefficient from the source to (*x*, *y*, *z*), *γ*_*x*,*y*,*z*_(*m*, *f*) is the transmission coefficient from (*x*, *y*, *z*) to the *m*th hydrophone, and *G*_*x*,*y*,*z*_ is the reflection coefficient of the insonified region at (*x*, *y*, *z*). *G*_*x*,*y*,*z*_ is also an occupancy indicator, i.e, ∣*G*_*x*,*y*,*z*_∣ = 0 if there is no object at (*x*, *y*, *z*). For simplification, we discretise our search space into *N* 3D cubical voxels, and approximate Eq. (1) by a summation over *N* voxels, as

2 $$x(m,f,k)= \sum\limits_{n=1}^{N}\zeta (f,k){G}_{n}{\nu }_{n}(k){\beta }_{n}(f,k){\gamma }_{n}(m,f) {\Delta}_x {\Delta}_y {\Delta}_z,$$

where subscript _*n*_ denotes the *n*th voxel, and the voxel spacings ∆*x*, ∆*y* and ∆*z* can be set to 1 without loss of generality. We can express *γ*_*n*_(*m*, *f*) as

3 $${\gamma }_{n}(m,f)={q}_{\gamma ,n}(m)\exp \left(-\frac{{{{{{{{\rm{i}}}}}}}}2\pi f{t}_{n}(m)}{c}\right),$$

where ${{{{{{{\rm{i}}}}}}}}=\sqrt{-1}$, *c* is the speed of sound in the pool water, and *t*_*n*_(*m*) is the distance from the *m*th sensor to the *n*th voxel. *q*_*γ*,*n*_(*m*) accounts for amplitude reduction due to the spreading of the wave propagating across this distance. We assume the object and the sensor array are far enough so that the received sound wave’s amplitude changes slowly across the sensors as compared to the phase. This is a reasonable assumption because in our experimental setups, the variability in *qγ,*_*n*_(*m*) across sensors is <5%. Thus, the variation of amplitude between sensor–voxel pairs can be neglected. Hence *q*_*γ*,*n*_(*m*) is approximately equal for all sensor–voxel pairs and is denoted as a constant *q*_*γ*_. Likewise, *β*_*n*_(*f*, *k*) is given by

4 $${\beta }_{n}(f,k)={q}_{\beta ,n}(k)\exp \left(-\frac{{{{{{{{\rm{i}}}}}}}}2\pi f{p}_{n}(k)}{c}\right)$$

where *p*_*n*_(*k*) is the distance from the transmitting source to the *n*th voxel during the *k*th click, and *q*_*β*,*n*_ accounts for the amplitude reduction due to spreading of the wave propagating across this distance. Again, we assume *p*_*n*_(*k*) is large enough that variability in *q*_*β*,*n*_(*k*) across clicks and voxels can be neglected and it is denoted as a constant *q*_*β*_.

Based on this generic model, we derive specific data models for the two cases—when the transmissions are (1) from the biomimetic-sonar and (2) from the dolphin-echolocation. In the biomimetic-sonar experiments, the transmit signal *ζ*(*f*, *k*) is predetermined by us. Hence, in this case, we matched-filter the recorded data with the transmit signal. Since the signal is broadband, its autocorrelation is a narrow pulse^58^. If we assume this to be an impulse with a peak *q*_*ζ*_, the spectral representation of the matched-filter output of the received signal at frequency *f*, sensor *m* and click *k* can be expressed as

5 $$y(m,f,k)=\mathop{\sum }\limits_{n=1}^{N}{\tilde{G}}_{n}(k)\exp \left(-\frac{{{{{{{{\rm{i}}}}}}}}2\pi f({p}_{n}(k)+{t}_{n}(m))}{c}\right)$$

where ${\tilde{G}}_{n}(k)={q}_{\zeta }{q}_{\beta }{q}_{\gamma }{G}_{n}{\nu }_{n}(k)$. Note that *G*_*n*_ = 0 or *ν*_*n*_(*k*) = 0 implies that ${\tilde{G}}_{n}(k)=0$. Hence ${\tilde{G}}_{n}(k)$ is an indicator of the presence of an object in the *n*^*th*^ voxel, provided the biomimetic-sonar’s transmit beam in the *k*th click is incident on it. The reason we employ three transmitters in the biomimetic-sonar can now be better understood in the context of this model—it is because some voxels may not be insonified in some clicks (*ν*_*n*_(*k*) = 0 for some *n* or *k*). So, we use multiple clicks with varying directivity/angles of insonification to ensure ${\tilde{G}}_{n}(k)$ is non-zero in at least some of the clicks for the occupied voxels.

The *M* × 1 matched-filtered output vector of the observed data at all sensors, at each click and frequency, is defined as **y**(*f*, *k*) = [*y*(1, *f*, *k*), *y*(2, *f*, *k*). . . , *y*(*M*, *f*, *k*)]^*T*^, where ^T^ denotes the matrix transpose. From Eq. (5), **y**(*f*, *k*) can be represented as a sum of the matched-filtered signal vector and contributions from ambient noise as

6 $${{{{{{{\bf{y}}}}}}}}(f,k)={{{{{{{\bf{A}}}}}}}}(f,k){{{{{{{\bf{g}}}}}}}}(k)+{{{{{{{\bf{v}}}}}}}}(f,k),$$

where the occupancy vector ${{{{{{{\bf{g}}}}}}}}(k)\in {{\mathbb{C}}}^{N\times 1}$ has *n*th element ${\tilde{G}}_{n}(k)$ and ${{{{{{{\bf{v}}}}}}}}(f,k)\in {{\mathbb{C}}}^{M\times 1}$ represents the effect of noise on the matched-filter output. The matrix ${{{{{{{\bf{A}}}}}}}}(f,k)\in {{\mathbb{C}}}^{M\times N}$ has *m*, *n*th element ${A}_{m,n}(f,k)=\exp \left(-\frac{i2\pi f({p}_{n}(k)+{t}_{n}(m))}{c}\right)$.

For the dolphin-echolocation data, only the sensor locations are known, whereas the transmit parameters such as the dolphin’s acoustic source position, transmit time and signal are unknown quantities. We deal with this lack of information by representing the model differently as compared to the previous case. We represent the received signal at each *m*th sensor as

7 $$x(m,f,k)=\mathop{\sum }\limits_{n=1}^{N}{\tilde{G}}_{n}(f,k)\exp \left(-\frac{{{{{{{{\rm{i}}}}}}}}2\pi f{t}_{n}(m)}{c}\right),$$

where ${\tilde{G}}_{n}(f,k)={q}_{\gamma }\zeta (f,k){G}_{n}{\nu }_{n}(k){\beta }_{n}(f,k)$. This encompasses our lack of knowledge of some signal parameters in addition to the voxels containing the object, clumped together. *G*_*n*_ = 0 or *ν*_*n*_(*k*) = 0 implies that ${\tilde{G}}_{n}(f,k)=0$. Hence ${\tilde{G}}_{n}(f,k)$ is an indicator of the presence of an object in the *n*th voxel, provided the dolphin’s transmit beam in the *k*th click is incident on it.

Based on the model in Eq. (7), we are in a better position to understand why not all object features are captured in the dolphin-echolocation data visualisation. One reason for this may be that Ginsan uses his beam directionality to insonify only some parts of the objects to get enough features to identify them (*ν*_*n*_(*k*) = 0 for some *n* or *k*), which is why only some parts are captured prominently. In addition, Ginsan mostly echolocates from a location vertically below the object. Thus, the shape features in the lower half of the object are closer to Ginsan during the echolocation, which may explain why only they are captured in all the outputs rather than the features in the top half. There are also distortions in some features such as a warping of the lower pipes of SQ and FF. This might be because the prominent target echoes come from points along the tube which are not necessarily in a straight line. Also, it is possible that we are not reconstructing the dolphin-echolocation visualisation as effectively as Ginsan because more information on the transmit signal is available to him.

From Eq. (7), the *M* × 1 observed data vector **x**(*f*, *k*) = [*x*(1, *f*, *k*), *x*(2, *f*, *k*). . . , *x*(*M*, *f*, *k*)]^*T*^ at all sensors, at each click and frequency can be represented as a sum of the received signal vector and the ambient noise, as

8 $${{{{{{{\bf{x}}}}}}}}(f,k)={{{{{{{\bf{B}}}}}}}}(f){{{{{{{\bf{g}}}}}}}}(f,k)+{{{{{{{\bf{w}}}}}}}}(f,k),$$

where the occupancy vector ${{{{{{{\bf{g}}}}}}}}(f,k)\in {{\mathbb{C}}}^{N\times 1}$ has *n*th element ${\tilde{G}}_{n}(f,k)$, ${{{{{{{\bf{w}}}}}}}}(f,k)\in {{\mathbb{C}}}^{M\times 1}$ represents the ambient noise component in the data, and the matrix ${{{{{{{\bf{B}}}}}}}}(f)\in {{\mathbb{C}}}^{M\times N}$ has *m*, *n*th element ${B}_{m,n}(f)=\exp \left(-\frac{{{{{{{{\rm{i}}}}}}}}2\pi f{t}_{n}(m)}{c}\right)$.

In order to use these models for visualising the data, we first fix the space to be scanned via the processor—a gridded 2D *x*–*y* cuboidal region spanned by the sample objects considered, with a grid spacing of 0.5 cm. Based on this, the matrices **A** and **B** can be constructed for the two scenarios based on the known information. The frequency-domain linear model developed above facilitates the use of convex optimisation methods to solve the acoustic imaging problem.

### Bartlett processing

We wish to interpret the echoes of the dolphin/biomimetic-sonar clicks to understand what acoustic information is present about the shape of the sample object. This involves inverting the acoustic data to estimate the occupancy vector **g** for the relevant frequencies and clicks used in the experiment.

In the biomimetic-sonar experiments, the transmit parameters are known to us. Hence, we can successfully reverse the phase change undergone by each frequency component of the echoes during propagation from the transmitter to the object to the sensors. We can scan for occupancy of the voxels in the search space by coherently using the information on phase variation across sensors and frequencies, by reversing the model-predicted phase changes undergone by the wave incident at each voxel. Using the Bartlett approach, we estimate the occupancy vector ${\hat{{{{{{{{\bf{g}}}}}}}}}}_{{{{{{{{\rm{B}}}}}}}}}$ for each click as^45^

9 $${\hat{{{{{{{{\bf{g}}}}}}}}}}_{{{{{{{{\rm{B}}}}}}}}}(k)=\sqrt{\mathop{\sum }\limits_{i=1}^{F}{{{{{{{\rm{diag}}}}}}}}\left({{{{{{{{\bf{A}}}}}}}}}^{{{{{{{{\rm{H}}}}}}}}}({f}_{i},k){{{{{{{\bf{y}}}}}}}}({f}_{i},k){{{{{{{{\bf{y}}}}}}}}}^{{{{{{{{\rm{H}}}}}}}}}({f}_{i},k){{{{{{{\bf{A}}}}}}}}({f}_{i},k)\right)},$$

where *f*_*i*_ is the frequency at the *i*th bin considered, *F* is the total number of frequency bins considered, diag(.) indicates a vector composed of the main-diagonal entries of its matrix argument, and ^H^ indicates the Hermitian transpose. The Bartlett processor output vector ${{{{{{{{\bf{h}}}}}}}}}_{{{{{{{{\rm{B}}}}}}}}}\in {{\mathbb{C}}}^{N}$ is obtained as the average of the outputs for all the *K* clicks, as

10 $${{{{{{{{\bf{h}}}}}}}}}_{{{{{{{{\rm{B}}}}}}}}}=\frac{1}{K}\mathop{\sum }\limits_{k=1}^{K}{\hat{{{{{{{{\bf{g}}}}}}}}}}_{{{{{{{{\rm{B}}}}}}}}}(k)$$

In the dolphin-echolocation trials, the transmit time of the click, transmit signal and position of the source are unknown to us. Thus, we do not have enough information to reverse the phase changes undergone by each frequency component of the wave during its propagation. This prevents us from combining the information across frequencies coherently. This lack of information is manifested in terms of the frequency-dependence of the unknown quantity **g**(*f*, *k*) for the dolphin-echolocation data case (Eq. (8)) which shows that we have *N* × 1 unknown variables at every frequency, as opposed to the biomimetic-sonar case where we had a single unknown vector for all frequencies.

Hence, for the dolphin-echolocation data, we cannot use coherent processing which combines acoustic phase information in the data across frequencies and provides better suppression of grating lobes and noise. This represents the disadvantage when the transmission parameters are unknown to the processor, and explains why Fig. 4 is qualitatively better than Fig. 5 and Supplementary Fig. 3. This limitation applies to both the Bartlett and SA approaches and has been highlighted in earlier works as well^59^. Thus, we resort to incoherent combinations across frequencies. To do this with the Bartlett approach, we first estimate the occupancy for each *k*th click and *i*th frequency as

11 $${\hat{{{{{{{{\bf{g}}}}}}}}}}_{{{{{{{{\rm{B}}}}}}}}}({f}_{i},k)=\sqrt{{{{{{{{\rm{diag}}}}}}}}\left({{{{{{{{\bf{B}}}}}}}}}^{{{{{{{{\rm{H}}}}}}}}}({f}_{i}){{{{{{{\bf{x}}}}}}}}({f}_{i},k){{{{{{{{\bf{x}}}}}}}}}^{{{{{{{{\rm{H}}}}}}}}}({f}_{i},k){{{{{{{\bf{B}}}}}}}}({f}_{i})\right)},$$

The Bartlett processor’s output vector is obtained as

12 $${{{{{{{{\bf{h}}}}}}}}}_{{{{{{{{\rm{B}}}}}}}}}=\frac{1}{FK}\mathop{\sum }\limits_{k=1}^{K}\mathop{\sum }\limits_{i=1}^{F}{\hat{{{{{{{{\bf{g}}}}}}}}}}_{{{{{{{{\rm{B}}}}}}}}}({f}_{i},k)$$

Notice that in this case, the magnitude (square root of energy) of the output is averaged across frequencies, representing the incoherent summation.

### Sparsity-aware processing

Conventional approaches such as Bartlett seek to answer the question: ‘How likely is this spatial region to contain a portion of the object?’. This question is tackled for each spatial region independent of other regions. In contrast, sparsity-aware processing answers a different question: ‘What is the minimum region occupied by the object that can explain the observed data?’. The SA solution to whether each region contains the object or not is obtained by considering the solution at other regions jointly.

In the biomimetic-sonar case, we can solve the inversion problem for **g**(*k*) if we formulate it as a minimisation problem given by

13 $$\begin{array}{rc}&{\hat{{{{{{{{\bf{g}}}}}}}}}}_{{{{{{{{\rm{SA}}}}}}}}}(k)=\mathop{{{{{{\rm{arg}}}}}}\;{{{{{\rm{min}}}}}}}\limits_{{{{{{{{\bf{g}}}}}}}}(k)}\parallel \!{{{{{{{\bf{g}}}}}}}}(k){\parallel }_{p}{{{{{{{\rm{subject \;to}}}}}}}}\\ &\left(\mathop{\sum }\limits_{i=1}^{F}\parallel \!{{{{{{{\bf{y}}}}}}}}({f}_{i},k)-{{{{{{{\bf{A}}}}}}}}({f}_{i},k){{{{{{{\bf{g}}}}}}}}(k){\parallel }_{2}^{2}\right) \; < \;\epsilon ,\end{array}$$

where ∥.∥_*p*_ denotes the vector *p*-norm. For values of *p* close to or less than 1, this minimisation enforces sparsity in the number of object-occupied (active) voxels in ${\hat{{{{{{{{\bf{g}}}}}}}}}}_{{{{{{{{\rm{SA}}}}}}}}}(k)$. To be more specific, the processor estimates **g**(*k*) with a sparse set of active voxels due to the minimisation of the *p*-norm across the columns, with *p* close to 1. Asserting **g**(*k*) to be frequency-independent and solving the problem for all frequencies simultaneously imposes consistency of the frequency information, as highlighted in ‘Discussion’. The inequality constraint forces the estimate to follow the observed data within a tolerance margin *ϵ* to account for noise. The choice of *ϵ* draws a trade-off between how well the estimated value ${\hat{{{{{{{{\bf{g}}}}}}}}}}_{{{{{{{{\rm{SA}}}}}}}}}(k)$ explains the data, versus how sparse it is. We set the value of *ϵ* to be a fraction of the total energy of the data, given by $\mathop{\sum }\nolimits_{i=1}^{F}\parallel \!{{{{{{{\bf{y}}}}}}}}({f}_{i},k){\parallel }_{2}^{2}$. The value of *p* also defines how sparse the output is expected to be, with smaller values of *p* yielding more sparsity. *p* is usually set to 1.05 in the biomimetic-sonar case.

For the case of the dolphin-echolocation data, in order to solve the problem, we define the *N* × *F* matrix ${{{{{{{\bf{G}}}}}}}}(k)=\left[{{{{{{{{\bf{g}}}}}}}}}_{{{{{{{{\rm{SA}}}}}}}}}({f}_{1},k),{{{{{{{{\bf{g}}}}}}}}}_{{{{{{{{\rm{SA}}}}}}}}}({f}_{2},k)...{{{{{{{{\bf{g}}}}}}}}}_{{{{{{{{\rm{SA}}}}}}}}}({f}_{F},k)\right]$ as the object occupancy matrix for the *k*th click. This matrix encompasses information on which portions of the scanned region reflected the echoes at any particular frequency. Using the SA approach, we formulate the inversion problem for **G**(*k*) as

14 $$\begin{array}{rc}&{\hat{{{{{{{{\bf{G}}}}}}}}}}_{{{{{{{{\rm{SA}}}}}}}}}(k)=\mathop{{{{{{\rm{arg}}}}}}\;{{{{{\rm{min}}}}}}}\limits_{{{{{{{{\bf{G}}}}}}}}(k)}\parallel \!{{{{{{{\bf{G}}}}}}}}(k){\parallel }_{p,2}{{{{{{{\rm{subject \;to}}}}}}}}\\ &\left(\mathop{\sum }\limits_{i=1}^{F}\parallel \!{{{{{{{\bf{x}}}}}}}}({f}_{i},k)-{{{{{{{\bf{B}}}}}}}}({f}_{i}){{{{{{{\bf{g}}}}}}}}({f}_{i},k){\parallel }_{2}^{2}\right) \, < \,\epsilon ,\end{array}$$

This formulation involves minimising a (*p*, 2)-norm in the cost function. Similar to the above case, for *p* close to 1, this minimisation enforces sparsity in the number of active rows, i.e., object-occupied voxels. However, within active rows, the columns may all be assigned occupancy values, which is consistent with the fact that the signal is broadband and thus there is expected to be energy at all frequencies considered (consistency information). We use *p* = 1 in this case (i.e. smaller than the biomimetic-sonar case). This is because the SNR is poorer, and the challenge posed by grating lobes is more in this case because coherent processing cannot be done across frequencies (see Bartlett processing section). Thus, in this case, we would like the output to focus more on suppressing the grating lobes and exhibiting the most relevant few features from the echoes which likely correspond to the target.

Now that we have represented the SA processing problem for the biomimetic-sonar and dolphin-echolocation cases in the forms presented in Eqs. (13) and (14), they can be solved efficiently using convex optimisation techniques because the cost functions and constraints of the formulations are convex for *p* ≥ 1. These particular types of problems that search for sparse solutions have been discussed in the compressed sensing literature, and the underlying mathematics has been tackled in earlier works^51,53^. We solve Eqs. (13) and (14) using the CVX toolbox^60^ and the MOSEK optimiser^61^ in MATLAB software.

For the biomimetic-sonar case, we solve three clicks (one click from each transmitter) when there is not much ambient noise. For the dolphin-echolocation case, we solve all the transmitted clicks identified.

We then obtain the SA processor output vector ${{{{{{{{\bf{h}}}}}}}}}_{{{{{{{{\rm{SA}}}}}}}}}\in {{\mathbb{C}}}^{N\times 1}$ for the biomimetic and dolphin-echolocation cases as

15 $${{{{{{{{\bf{h}}}}}}}}}_{{{{{{{{\rm{SA}}}}}}}}}=\frac{1}{K}\mathop{\sum }\limits_{k=1}^{K}| {\hat{{{{{{{{\bf{g}}}}}}}}}}_{{{{{{{{\rm{SA}}}}}}}}}(k)| ,$$

and

16 $${{{{{{{{\bf{h}}}}}}}}}_{{{{{{{{\rm{SA}}}}}}}}}=\frac{1}{FK}\mathop{\sum }\limits_{k=1}^{K}\mathop{\sum }\limits_{i=1}^{F}| {\hat{{{{{{{{\bf{g}}}}}}}}}}_{{{{{{{{\rm{SA}}}}}}}}}({f}_{i},k)| ,$$

respectively, where ${\hat{{{{{{{{\bf{g}}}}}}}}}}_{{{{{{{{\rm{SA}}}}}}}}}({f}_{i},k)$ indicates the *i*th column of ${\hat{{{{{{{{\bf{G}}}}}}}}}}_{{{{{{{{\rm{SA}}}}}}}}}(k)$. We plot all the processor outputs with the lower and upper limits of the colour scale set at 5th and 99.5th percentiles of the output voxel values respectively.

### Computation of discrimination coefficient

We correlate the processor outputs against visual representations of the sample and alternative objects using matched filtering^57^. First, we compute the matched-filter templates corresponding to the SQ and FF objects as binary masks ${{{{{{{{\bf{s}}}}}}}}}_{i}\in {{\mathbb{C}}}^{N\times 1}$ where subscript *i* ∈ {SQ, FF} denotes the object correlated against. In these masks, voxels that are occupied by the object have an entry of 1, and voxels that are not occupied by the object have an entry of 0. The spatial extent of the masks is the same as the region scanned by the processor. Then, the matched-filter correlation of the processor output with the two templates are computed and normalised with respect to their 2-norms, expressed as

17 $${C}_{i}=\frac{{{{{{{{{\bf{h}}}}}}}}}^{{{{{{{{\rm{T}}}}}}}}}{{{{{{{{\bf{s}}}}}}}}}_{i}}{\parallel \!{{{{{{{\bf{h}}}}}}}}{\parallel }_{2}.\parallel \!{{{{{{{{\bf{s}}}}}}}}}_{i}{\parallel }_{2}}$$

*C*_*i*_ measures the degree of overlap with the object *i*. We then compute the ratio *r* of the matched-filter correlation with the correct alternative’s template versus the wrong alternative’s template, as $r=\frac{{C}_{{{{{{{{\rm{SQ}}}}}}}}}}{{C}_{{{{{{{{\rm{FF}}}}}}}}}}$ if the sample object is SQ, and $r=\frac{{C}_{{{{{{{{\rm{FF}}}}}}}}}}{{C}_{{{{{{{{\rm{SQ}}}}}}}}}}$ if the sample object is FF. The discrimination coefficient *R* is defined as *r* converted to decibels, i.e.

18 $$R=20{\log }_{10}(r).$$

### Reporting summary

Further information on research design is available in the Nature Research Reporting Summary linked to this article.

### Supplementary information

Description of Additional Supplementary Files

Supplementary information

Supplementary Movie 1

Supplementary Movie 2

Reporting Summary

## Data Availability

Processed acoustic data used in the study is available at the online repository Zenodo at 10.5281/zenodo.6413159^46^.

## Code availability

MATLAB Code to visualise the acoustic data used in the study is available at the online repository Zenodo at 10.5281/zenodo.6413159^46^.

## References

[1] Blondel, P. *The Handbook of Sidescan Sonar* (Springer Berlin Heidelberg, 2009).

[2] Roitblat, H., Au, W., Nachtigall, P., Shizumura, R. & Moons, G. Sonar recognition of targets embedded in sediment. *Neural Networks* **8**, 1263–1273 (1995). 10.1016/0893-6080(95)00052-6

[3] Moore, P. W. Mine-hunting dolphins of the Navy. In *SPIE 3079, Detection and Remediation Technologies for Mines and Minelike Targets II*. 10.1117/12.280845 (1997).

[4] Au, W. History of dolphin biosonar research. *Acoust. Today* **11**, 4–7 (2015).

[5] DeLong, C. M., Au, W. W. L., Lemonds, D. W., Harley, H. E. & Roitblat, H. L. Acoustic features of objects matched by an echolocating bottlenose dolphin. *J. Acoust. Soc. Am.* **119**, 1867–1879 (2006). 16583925 10.1121/1.2161434

[6] Pack, A. A. & Herman, L. M. Sensory integration in the bottlenosed dolphin: immediate recognition of complex shapes across the senses of echolocation and vision. *J. Acoust. Soc. Am.* **98**, 722–733 (1995). 7642811 10.1121/1.413566

[7] Herman, L. M., Pack, A. A. & Hoffmann-Kuhnt, M. Seeing through sound: dolphins (*Tursiops truncatus*) perceive the spatial structure of objects through echolocation. *J. Comparative Psychol.* **112**, 292–305 (1998). 10.1037/0735-7036.112.3.292 9770316

[8] Hoffmann-Kuhnt, M. et al. Is synthetic aperture an essential tool for echoic shape recognition in dolphins? In OCEANS’11 MTS/IEEE KONA, 1-7 (IEEE, Waikoloa, HI, 2011).

[9] Hoffmann-Kuhnt, M., Chitre, M., Mátrai, E., Yeo, K. & Lee, J. Dolphin echolocation—synthetic aperture or “raster scanning”? *J. Acoust. Soc. Am.* **131**, 3362–3362 (2012). 10.1121/1.4708669

[10] Wei, C. et al. Possible limitations of dolphin echolocation: a simulation study based on a cross-modal matching experiment. *Sci. Rep.* **11**, 1–14 (2021). 33758216 10.1038/s41598-021-85063-2 PMC7988039

[11] Au, W. W. L. *The Sonar of Dolphins* (Springer New York, 1993).

[12] Paihas, Y., Capus, C., Brown, K. & Lane, D. Benefits of dolphin inspired sonar for underwater object identification. In *Biomimetic and Biohybrid Systems* (eds Lepora, N. F., Mura, A., Krapp, H. G., Verschure, P. F. M. J. & Prescott, T. J.), 36–46 (Springer Berlin Heidelberg, 2013).

[13] Kloepper, L. N., Nachtigall, P. E., Donahue, M. J. & Breese, M. Active echolocation beam focusing in the false killer whale, *Pseudorca crassidens*. *J. Exp. Biol.* **215**, 1306–1312 (2012). 22442368 10.1242/jeb.066605

[14] Olivieri, M. P. Bio-inspired broadband SONAR technology for small UUVs. *Oceans Conference Record (IEEE)* **4**, 2135–2144 (2002).

[15] Brill, R. L., Sevenich, M. L., Sullivan, T. J., Sustman, J. D. & Witt, R. E. Behavioral evidence for hearing through the lower jaw by an echolocating dolphin (*Tursiops Truncatus*). *Marine Mammal Sci.* **4**, 223–230 (1988). 10.1111/j.1748-7692.1988.tb00203.x

[16] Ketten, D. Functional analyses of whale ears: adaptations for underwater hearing. in *Proceedings of OCEANS’94,* Vol. 1, I/264–I/270 (IEEE, 2015).

[17] Cranford, T. W., Krysl, P. & Hildebrand, J. A. Acoustic pathways revealed: simulated sound transmission and reception in Cuvier’s beaked whale (*Ziphius cavirostris*). *Bioinspiration Biomimetics* **3**, 016001 (2008). 10.1088/1748-3182/3/1/016001 18364560

[18] Song, Z. et al. Investigation on acoustic reception pathways in finless porpoise (*Neophocaena asiaorientalis* sunameri) with insight into an alternative pathway. *Bioinspiration Biomimetics* **14.1**, 016004 (2018). 10.1088/1748-3190/aaeb01 30421726

[19] Popov, V. V., Nechaev, D. I., Sysueva, E. V. & Supin, A. Y. Level-dependent masking of the auditory evoked responses in a dolphin: manifestation of the compressive nonlinearity. *J. Comparative Physiol. A: Neuroethol., Sensory, Neural, Behav. Physiol.* **205**, 839–846 (2019). 10.1007/s00359-019-01370-0 31555834

[20] Au, W. W. L., Fay, R. R. & Popper, A. N. (eds.) Hearing by whales and dolphins, Vol. 12. in *Springer Handbook of Auditory Research* (Springer New York, 2000).

[21] Houser, D. S., Helweg, D. A. & Moore, P. W. Classification of dolphin echolocation clicks by energy and frequency distributions. *J. Acoust. Soc. Am.* **106**, 1579–1585 (1999). 10489713 10.1121/1.427153

[22] Johnson, D. H. & Dudgeon, D. E. *Array Signal Processing: Concepts and Techniques* (Simon & Schuster, Inc., 1992).

[23] Branstetter, B. K., Mevissen, S. J., Herman, L. M., Pack, A. A. & Roberts, S. P. Horizontal angular discrimination by an echolocating bottlenose dolphin *Tursiops truncatus*. *Bioacoustics* **14**, 15–34 (2003). 10.1080/09524622.2003.9753510

[24] Capus, C. et al. Bio-inspired wideband sonar signals based on observations of the bottlenose dolphin (*Tursiops truncatus*). *J. Acoust. Soc. Am.* **121**, 594–604 (2007). 17297813 10.1121/1.2382344

[25] Gaudette, J. E., Donskoy, D. M., Martin, C. J., Murphy, C. T. & Simmons, J. A. Bio-inspired broadband sonar array prototypes and underwater experiments for two- and three-dimensional acoustic imaging applications. *J. Acoust. Soc. Am.* **140**, 3033–3033 (2016). 10.1121/1.4969409

[26] Benoit-Bird, K. J., Au, W. W. L. & Kelley, C. D. Acoustic backscattering by Hawaiian lutjanid snappers. I. Target strength and swimbladder characteristics. *J. Acoust. Soc. Am.* **114**, 2757 (2003). 14650010 10.1121/1.1614256

[27] Benoit-Bird, K. J., Au, W. W., Kelley, C. D. & Taylor, C. Acoustic backscattering by deepwater fish measured in situ from a manned submersible. *Deep-Sea Research Part I: Oceanographic Res. Papers* **50**, 221–229 (2003). 10.1016/S0967-0637(02)00160-7

[28] Au, W. W. L. & Benoit-Bird, K. J. Acoustic backscattering by Hawaiian lutjanid snappers. II. Broadband temporal and spectral structure. *J. Acoust. Soc. Am.* **114**, 2767 (2003). 14650011 10.1121/1.1614257

[29] Au, W. W. L., Benoit-Bird, K. J. & Kastelein, R. A. Modeling the detection range of fish by echolocating bottlenose dolphins and harbor porpoises. *J. Acoust. Soc. Am.* **121**, 3954 (2007). 17552742 10.1121/1.2734487

[30] Pailhas, Y., Capus, C., Brown, K. & Moore, P. Analysis and classification of broadband echoes using bio-inspired dolphin pulses. *J. Acoust. Soci. Am.* **127**, 3809–3820 (2010). 10.1121/1.3372754 20550279

[31] Pailhas, Y., Capus, C. & Brown, K. Bio-inspired sonar. in *2011 17th International Conference on Digital Signal Processing (DSP)*, 1–6 (IEEE, 2011).

[32] Imaizumi, T., Furusawa, M., Akamatsu, T. & Nishimori, Y. Measuring the target strength spectra of fish using dolphin-like short broadband sonar signals. *J. Acoust. Soc. Am.* **124**, 3440–3449 (2008). 19206773 10.1121/1.2990703

[33] Zhang, Y. et al. A biomimetic projector with high subwavelength directivity based on dolphin biosonar. *Appl. Phys. Lett.* **105**, 1–5 (2014).

[34] Gao, X. et al. Acoustic beam control in biomimetic projector via velocity gradient. *Appl. Phys. Lett.* **109**, 013505 (2016).

[35] Dong, E. et al. Physical modeling and validation of porpoises’ directional emission via hybrid metamaterials. *Natl Sci. Rev.* **6**, 921–928 (2019). 34691953 10.1093/nsr/nwz085 PMC8291406

[36] Dong, E. et al. Bioinspired metagel with broadband tunable impedance matching. *Sci. Adv.* **6**, 1–10 (2020). 10.1126/sciadv.abb3641 PMC7608802 33127672

[37] Song, Z. et al. Physical implementation of dolphin biosonar to facilitate ultrasound control. *Appl. Phys. Lett.* **117**, 173701 (2020).

[38] Song, Z., Zhang, C., Zhang, J., Ou, W. & Zhang, Y. A physical realization of porpoise biosonar concerning sound reception. *Appl. Phys. Lett.* **119**, 094103 (2021).

[39] Cheong, Y., Shorter, K. A. & Popa, B.-I. Acoustic scene modeling for echolocation in bottlenose dolphin. *J. Acoust. Soc. Am.* **150**, A121–A121 (2021). 10.1121/10.0007837

[40] Pack, A. A., Herman, L. M., Hoffmann-Kuhnt, M. & Branstetter, B. K. The object behind the echo: Dolphins (Tursiops truncatus) perceive object shape globally through echolocation. *Behav. Processes* **58**, 1–26 (2002). 11955768 10.1016/S0376-6357(01)00200-5

[41] Herman, L. M. What laboratory research has told us about dolphin cognition. *Int. J. Comparative Psychol.* **23**, 310–330 (2010). 10.46867/IJCP.2010.23.03.07

[42] Finneran, J. J. et al. High-resolution measurement of a bottlenose dolphin’s (*Tursiops truncatus*) biosonar transmission beam pattern in the horizontal plane. *J. Acoust. Soc. Am.* **136**, 2025–2038 (2014). 25324101 10.1121/1.4895682

[43] Kloepper, L. N. et al. Support for the beam focusing hypothesis in the false killer whale. *J. Exp. Biol.* https://journals.biologists.com/jeb/article/doi/10.1242/jeb.119966/258180/Support-for-the-beam-focusing-hypothesis-in-the (2015). 10.1242/jeb.119966 26056247

[44] Finneran, J. J., Mulsow, J., Branstetter, B., Moore, P. & Houser, D. S. Nearfield and farfield measurements of dolphin echolocation beam patterns: no evidence of focusing. *J. Acoust. Soc. Am.* **140**, 1346 (2016). 27586761 10.1121/1.4961015

[45] Baggeroer, A. B., Kuperman, W. A. & Schmidt, H. Matched field processing: source localization in correlated noise as an optimum parameter estimation problem. *J. Acoust. Soc. Am.* **83**, 571–587 (1988). 10.1121/1.396151

[46] Vishnu, H., Hoffmann-Kuhnt, M., Chitre, M., Ho, A. & Matrai, E. Data and code routines for biosonar data (Version 1) [Data set]. *Zenodo*10.5281/zenodo.6413159 (2022).

[47] Peters, B. & Kriegeskorte, N. Capturing the objects of vision with neural networks. *Nat. Hum. Behav.* **5**, 1127–1144 (2021). 34545237 10.1038/s41562-021-01194-6

[48] Seriès, P. & Seitz, A. R. Learning what to expect (in visual perception). *Front. Human Neurosci.* **7**, 668 (2013). 10.3389/fnhum.2013.00668 PMC3807544 24187536

[49] Morgenstern, Y., Murray, R. F. & Harris, L. R. The human visual system’s assumption that light comes from above is weak. *Proc. Natl Acad. Sci. USA* **108**, 12551–12553 (2011). 21746935 10.1073/pnas.1100794108 PMC3145687

[50] Zoeller, A. C., Lezkan, A., Paulun, V. C., Fleming, R. W. & Drewing, K. Integration of prior knowledge during haptic exploration depends on information type. *J. Vision* **19**, 20 (2019). 10.1167/19.4.20 30998830

[51] Donoho, D. Compressed sensing. *IEEE Transact. Inf. Theory* **52**, 1289–1306 (2006). 10.1109/TIT.2006.871582

[52] Eldar, Y & Kutyniok, G. *Compressed Sensing: Theory and Applications* (Cambridge University Press, 2012).

[53] Stojnic, M., Parvaresh, F. & Hassibi, B. On the reconstruction of block-sparse signals with an optimal number of measurements. *IEEE Transact. Sig. Processing* **57**, 3075–3085 (2009). 10.1109/TSP.2009.2020754

[54] Callaghan Innovation. TT-0006 Split beam Transducer SB-120-9. https://rd.callaghaninnovation.govt.nz/assets/Uploads/TT-0006-Split-beam-Transducer-SB-120-9-Datasheet.pdf (2021).

[55] Reson. Reson TC4013. http://www.teledynemarine.com/reson-tc4013 (2021).

[56] National Instruments. NI PXIe-1062Q. https://www.ni.com/en-sg/support/model.pxie-1062q.html (2021).

[57] Kay, S. M. *Fundamentals of Statistical Signal Processing, Vol. II: Detection Theory* (Prentice-Hall, 1998).

[58] Levanon, N. & Mozeson, E. *Radar Signals* (John Wiley & Sons Ltd, 2004).

[59] Edelmann, G. & Gaumond, C. Beamforming using compressive sensing. *J. Acoust. Soc. Am.* **130**, EL232 (2011). 21974497 10.1121/1.3632046

[60] Grant, M. & Boyd, S. CVX: Matlab software for disciplined convex programming, version 2.0 beta. http://cvxr.com/cvx (2013).

[61] MOSEK ApS. MOSEK Optimization Toolbox for MATLAB 9.2.42. https://docs.mosek.com/9.2/toolbox/index.html (2015).

