# Supplementary material for: A dolphin-inspired compact sonar for underwater acoustic imaging
Source: Commun Eng. 2022 Jun 8;1:10. doi: 10.1038/s44172-022-00010-x (PMC11341816; doi:10.1038/s44172-022-00010-x)
Supplement: Supplementary file 1 — Description of Additional Supplementary Files [file 44172_2022_10_MOESM1_ESM.pdf]

Description of Additional Supplementary Files

File Name: Supplementary Movie 1

Description: Example of a 2-alternative EV-MTS trial conducted in Ocean Park described in Methods, showing the dolphin entering the pool, echolocating on the sample object (SQ) and picking his choice correctly from the visual alternatives

File Name: Supplementary Movie 2

Description: Underwater video of EV-MTS trial showing the dolphin entering the arena, echolocating on the sample object (SQ) and exiting to pick his choice from visual alternatives
