# Supplementary material for: A dolphin-inspired compact sonar for underwater acoustic imaging
Source: Commun Eng. 2022 Jun 8;1:10. doi: 10.1038/s44172-022-00010-x (PMC11341816; doi:10.1038/s44172-022-00010-x)
Supplement: Supplementary file 2 — Supplementary information [file 44172_2022_10_MOESM2_ESM.pdf]

A dolphin-inspired compact sonar for underwater acoustic imaging.

Hari Vishnu [*,†,‡], Matthias Hoffmann-Kuhnt [†,‡], Mandar Chitre [†,‡], Abel Ho [†], Eszter Matrai [§]

[*] harivishnu@gmail.com

[†] Acoustic Research Laboratory, Tropical Marine Science Institute, 18 Kent Ridge Road, National University of Singapore, Singapore 119227.

[‡] Hari Vishnu, Matthias Hoffmann-Kuhnt and Mandar Chitre contributed equally to this work

[§] Ocean Park Hong Kong, 180 Wong Chuk Hang Road, Aberdeen, Hong Kong (SAR), China.

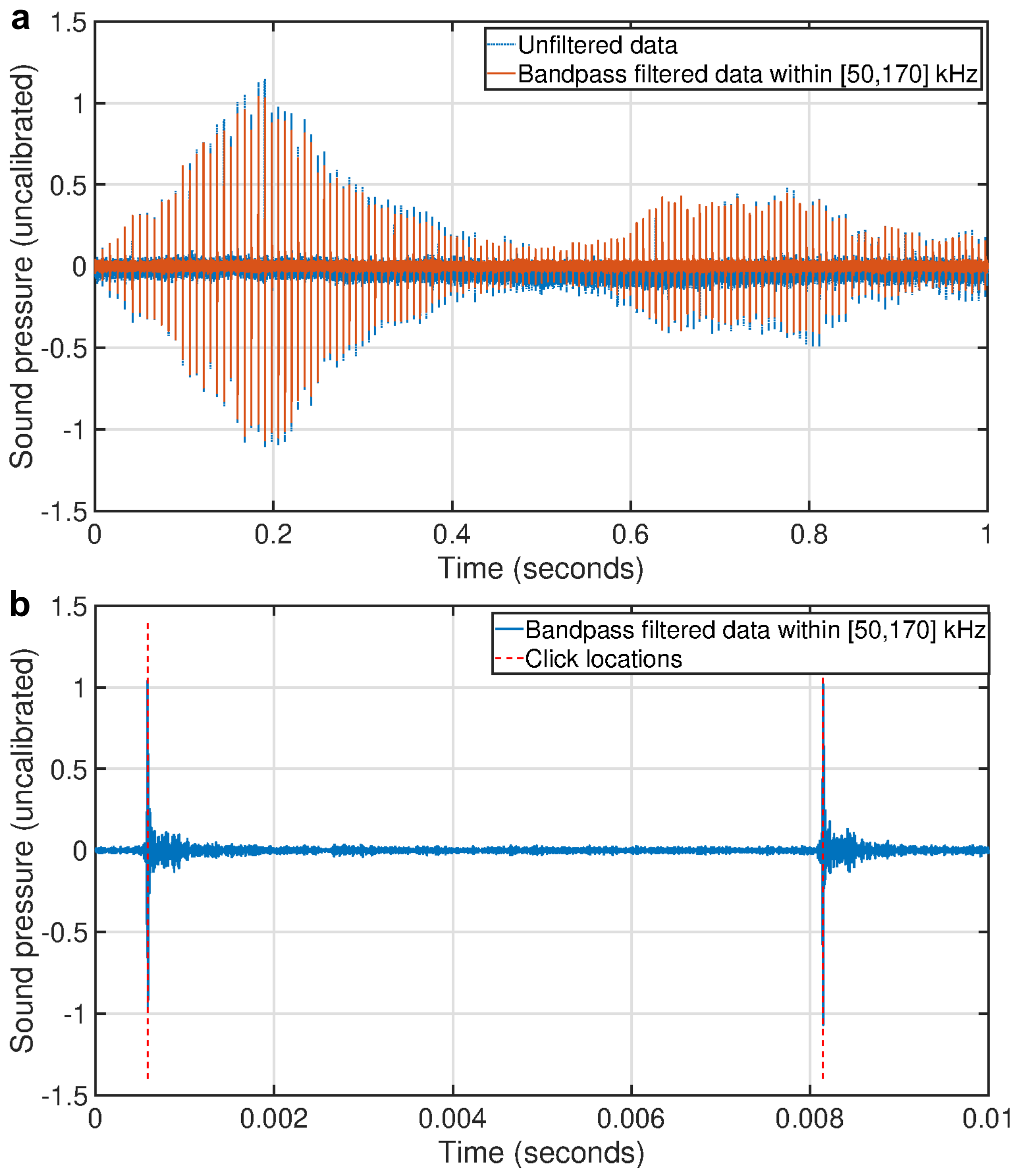

Supplementary Figure 1. Timeseries of recorded acoustic data. The timeseries shows the dolphin's transmitted clicks at a rate of ∼130 Hz, and return echoes from objects. **a,** 1 second of the acoustic data recorded at one sensor from dataset #1 showing the unfiltered data (blue) and bandpass filtered data within [50, 170] kHz (orange). The reduction in noise floor due to filtering is visible, and the reduction in signal amplitudes is nominal. **b,** Magnified image of the acoustic data (blue) and the transmit click timings identified by thresholding (red dashed lines). The two large peaks correspond to the transmit clicks. The increased energy in the period that follows them corresponds to the return echoes.

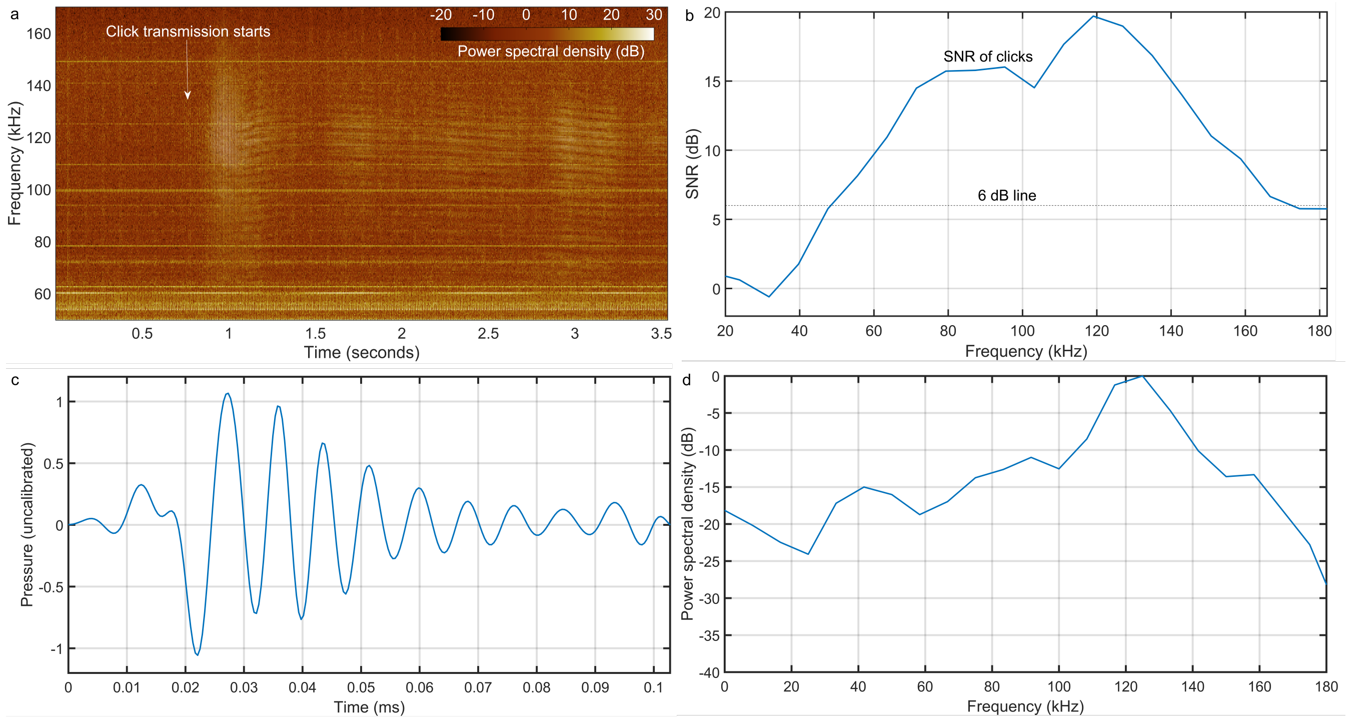

Supplementary Figure 2. Spectral analysis of recorded dolphin clicks and biomimetic transmitter signals showing their broadband nature. a, Spectrogram (in dB, median-normalised) of 3.5 seconds of dolphin acoustic data recorded at one sensor from EV-MTS dataset #1. The vertical lines correspond to broadband transmit clicks whose energy is spread across the spectrum. There is a variation in the frequency content of the clicks as the interrogation progresses. The stable horizontal lines observed are due to electronic noise. **b,** SNR (in dB) of the dolphin clicks recorded in dataset #1, averaged across all sensors. For transmit click identification, we use the band 50-170 kHz, which is where the average transmit click SNR is greater than 6 dB (denoted by the dashed line). **c,** Timeseries of transmit-signal used in the biomimetic transmitter. **d,** Power spectral density (in dB, normalised to peak) of the transmit-signal, showing the acoustic energy is concentrated in the 110-130 kHz band with the highest peak at 120 kHz, similar to that of the recorded dolphin clicks.

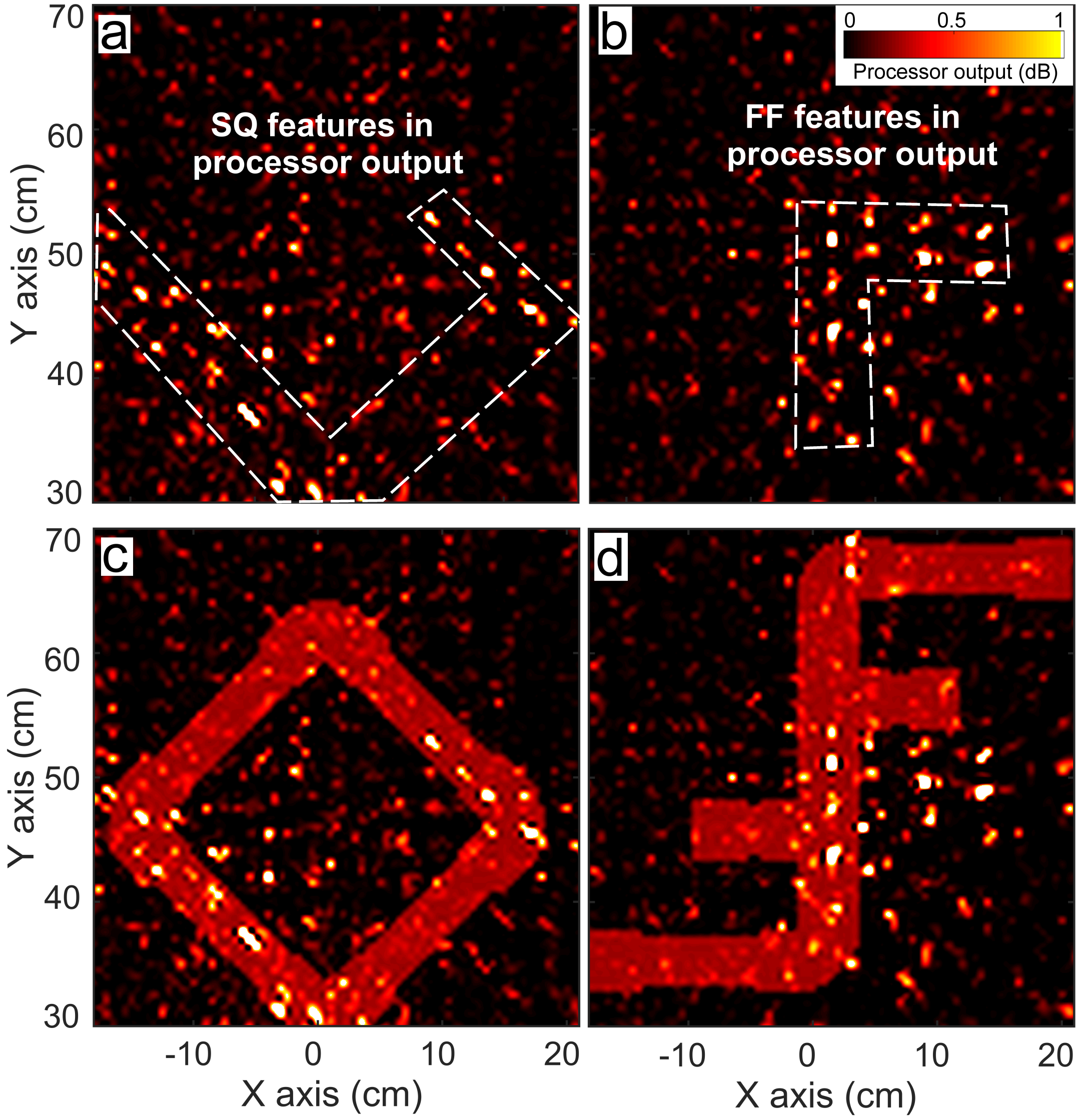

Supplementary Figure 3. SA processor visualisations exhibit shape features of the sample object. **a, b** Outputs using datasets #3 and #4 where SQ and FF are the samples respectively. **c, d,** Subfigures (a) and (b) shown with superposed shading masks in the shape of the respective sample, highlighting features matching the sample object's shape. These features are picked up by the matched-filter, yielding R_{SA} values of 1.26 dB and 1.85 dB respectively which indicate that the acoustic data contains enough information to identify the sample.

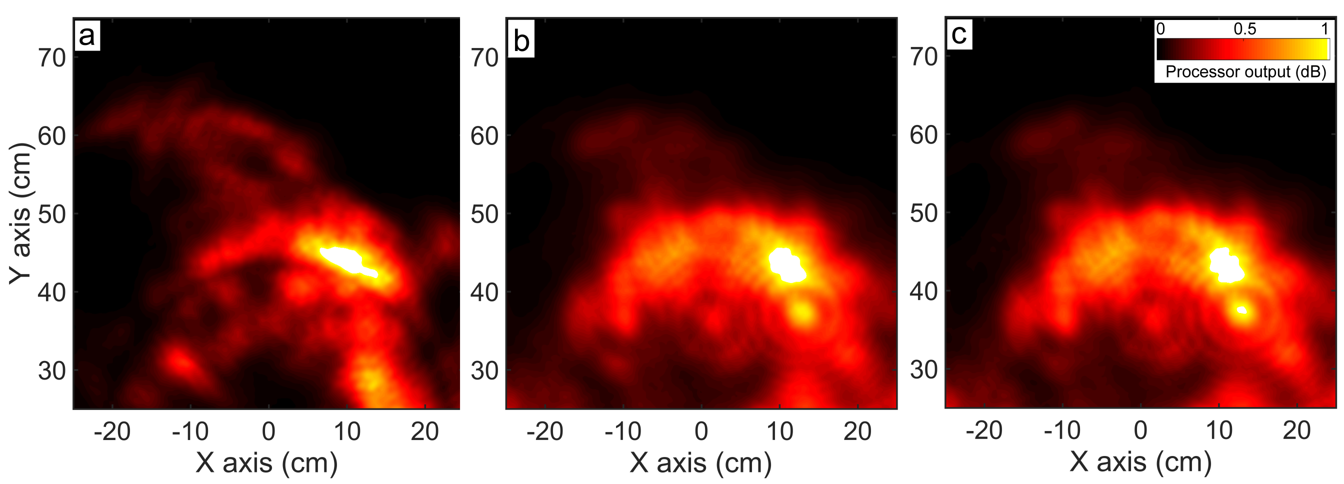

Supplementary Figure 4. SA processor visualisations with datasets #8 using different transmitter clicks. a, Output using the click from only the first transmitter shows the EL object features clearly. **b,** Output using all three transmitter clicks. The 'trunk' (drooping horizontal bar on the right) is more prominently seen, whereas other EL shape features are not as clear as (a), and are masked by strong echoes received from the trunk during the second and third transmitter clicks, which is shown in **c,** Output using returns from second and third transmitter clicks alone in dataset #8.

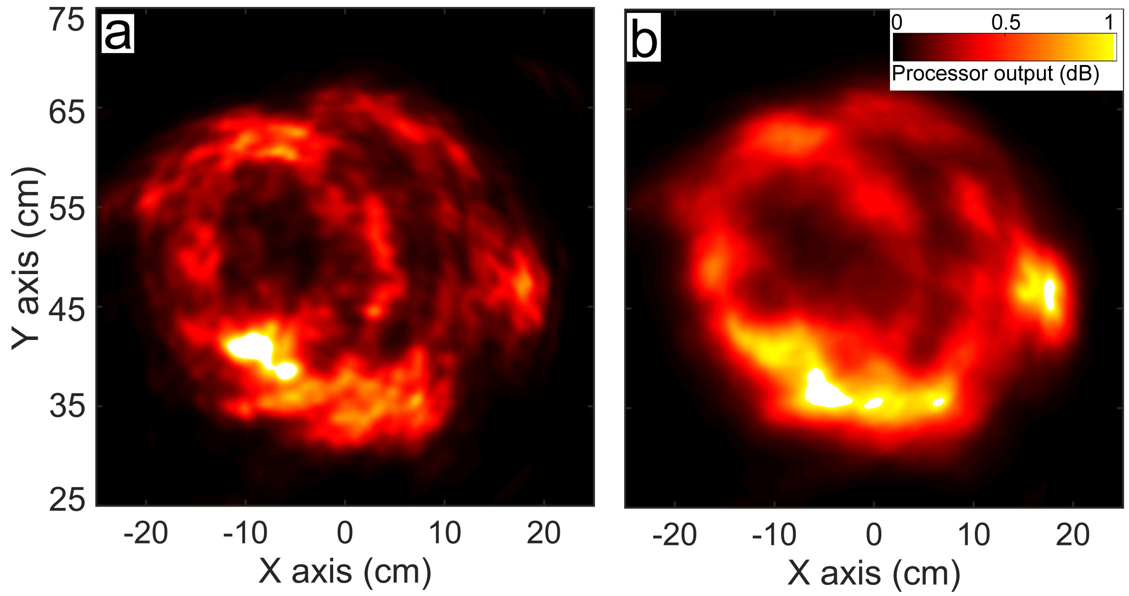

Supplementary Figure 5. Improvement in biomimetic sonar output quality due to use of multiple clicks. SA processor output with noisy version of biomimetic sonar dataset #5 with SNR of 20 dB. Comparison of processing done using **a**), three clicks (one per transmitter) and **b,** 60 clicks (20 per transmitter), highlights that in the presence of noise, clarity of imaging can be improved by using the repeated interrogation feature of the sonar. The output in **b** also yields superior target-discrimination performance with $R_{SA} = 4.03$ dB, which is more than the value of $R_{SA} = 2.77$ dB obtained for **a**.
