# Supplementary material for: A dolphin-inspired compact sonar for underwater acoustic imaging
Source: Commun Eng. 2022 Jun 8;1:10. doi: 10.1038/s44172-022-00010-x (PMC11341816; doi:10.1038/s44172-022-00010-x)
Supplement: Supplementary file 5 — Reporting Summary [file 44172_2022_10_MOESM5_ESM.pdf]

nature research

Corresponding author(s): Hari Vishnu

Last updated by author(s): Apr 4, 2022

Reporting Summary

Nature Research wishes to improve the reproducibility of the work that we publish. This form provides structure for consistency and transparency in reporting. For further information on Nature Research policies, see our Editorial Policies and the Editorial Policy Checklist.

Statistics

For all statistical analyses, confirm that the following items are present in the figure legend, table legend, main text, or Methods section.

n/a	Confirmed	
☐	☒	The exact sample size (n) for each experimental group/condition, given as a discrete number and unit of measurement
☐	☒	A statement on whether measurements were taken from distinct samples or whether the same sample was measured repeatedly
☐	☒	The statistical test(s) used AND whether they are one- or two-sided<br>*Only common tests should be described solely by name; describe more complex techniques in the Methods section.*
☐	☒	A description of all covariates tested
☒	☐	A description of any assumptions or corrections, such as tests of normality and adjustment for multiple comparisons
☒	☐	A full description of the statistical parameters including central tendency (e.g. means) or other basic estimates (e.g. regression coefficient) AND variation (e.g. standard deviation) or associated estimates of uncertainty (e.g. confidence intervals)
☐	☒	For null hypothesis testing, the test statistic (e.g. F, t, r) with confidence intervals, effect sizes, degrees of freedom and P value noted<br>*Give P values as exact values whenever suitable.*
☒	☐	For Bayesian analysis, information on the choice of priors and Markov chain Monte Carlo settings
☒	☐	For hierarchical and complex designs, identification of the appropriate level for tests and full reporting of outcomes
☒	☐	Estimates of effect sizes (e.g. Cohen's d, Pearson's r), indicating how they were calculated

Our web collection on statistics for biologists contains articles on many of the points above.

Software and code

Policy information about availability of computer code

Data collection	MATLAB was used to record the acoustic data
Data analysis	MATLAB v2020 was used for all acoustic data analysis. Python Scipy package was used for the statistical analysis.

For manuscripts utilizing custom algorithms or software that are central to the research but not yet described in published literature, software must be made available to editors and reviewers. We strongly encourage code deposition in a community repository (e.g. GitHub). See the Nature Research guidelines for submitting code & software for further information.

Data

Policy information about availability of data

All manuscripts must include a data availability statement. This statement should provide the following information, where applicable:

- Accession codes, unique identifiers, or web links for publicly available datasets
- A list of figures that have associated raw data
- A description of any restrictions on data availability

Acoustic data and associated plotting code are available via the free repository Zenodo at the link https://doi.org/10.5281/zenodo.6413159

Field-specific reporting

Please select the one below that is the best fit for your research. If you are not sure, read the appropriate sections before making your selection.

☒ Life sciences ☐ Behavioural & social sciences ☐ Ecological, evolutionary & environmental sciences

For a reference copy of the document with all sections, see nature.com/documents/nr-reporting-summary-flat.pdf

Life sciences study design

All studies must disclose on these points even when the disclosure is negative.

Sample size	Sample sizes were selected by ensuring they were large enough to get statistically significant results. For 4-alternative EV-MTS trials, if we wanted to look at a scenario where the dolphin performs correctly in more than 10 trials (i.e >=11), then from the binomial testing, we can determine that we need to perform atleast 20 trials to test if the dolphin's performance is significantly better than chance at a threshold level of p = 0.01. At the same time, the number of trials is limited by the operational hours allowed at the facility, and the possibility of fatiguing the dolphin by performing too many trials and affecting his performance. So 20 trials gives a sweet spot considering these two factors. Further, having the number of trials as a factor of 4 allows us to select pseudorandom configurations of the object such that each object is the sample an equal number of times, and all the 4 locations of the visual alternatives would have the correct choice an equal number of times.
Data exclusions	No data were excluded from the analysis - trials conducted on 2nd and 5th September 2014 (in which SQ or FF objects were used) were considered, since these were the objects focused on in the study. The other objects used in the 4-alternative EV-MTS trials were more complex and harder to analyse using the methods developed in this study, primarily because they had more depth (3d nature) than FF and SQ.
Replication	The operators who revealed the samples and alternatives were not the same as the trainer, thus ensuring objectivity in the trials, and minimising any bias or possibility of human cueing to the dolphin (more details are covered under blinding and randomization below). Furthermore, in the 20 trials, each object was repeated multiple times, and the results indicate the repeatability of the results across these trials (the dolphin got 6/6 when the objects were SQ or FF). We conducted more EV-MTS trials later on with the dolphin and have found that these results are highly repeatable in terms of good matching performance, but since the studies were testing different aspects or hypotheses, they are not included in this study, and these results are yet to be published.
Randomization	Several steps were taken to randomize the objects across the trials: During the 20 trials, the alternatives were all placed in different configurations using a prepared pseudo-random configuration, and the each of the four locations had the correct choice an equal number of times. The samples presented to the dolphin were also selected using a pseudo-random configuration, which ensured that the same sample did not repeat consecutively, in order to reduce recency bias on part of the dolphin. The sample configurations during sessions also ensured that all objects the study were repeated an equal number of times, thus ensuring it was a balanced study, in order to avoid biases due to data imbalance.
Blinding	During the interval in which the sample and alternative stimuli were changed, the dolphin stayed in an adjacent pool, thus preventing him from seeing the objects placed inside the boxes or prematurely echolocating on the next sample. Similarly, to ensure blind and unbiased trials, neither did the person operating the sample box know what alternatives had been placed in the boxes, nor did the person operating the alternative display boxes have any knowledge of the sample object. The trainer of the dolphin also stayed at the entrance of the pool and did not interact with the dolphin until the trial was over. Moreover, the box setups used ensured that no unintentional cueing of the dolphin was possible. An external researcher would then announce if the dolphin had made a correct choice and the trainer would reinforce the animals with a whistle and a fish.

Reporting for specific materials, systems and methods

We require information from authors about some types of materials, experimental systems and methods used in many studies. Here, indicate whether each material, system or method listed is relevant to your study. If you are not sure if a list item applies to your research, read the appropriate section before selecting a response.

Materials & experimental systems

n/a	Involved in the study	
☒	☐	Antibodies
☒	☐	Eukaryotic cell lines
☒	☐	Palaeontology and archaeology
☐	☒	Animals and other organisms
☒	☐	Human research participants
☒	☐	Clinical data
☒	☐	Dual use research of concern

Methods

n/a	Involved in the study	
☒	☐	ChIP-seq
☒	☐	Flow cytometry
☒	☐	MRI-based neuroimaging

Animals and other organisms

Policy information about studies involving animals; ARRIVE guidelines recommended for reporting animal research

Laboratory animals	Tursiops aduncus, male, age about 6 years at the time of the study. Subject was born in captivity.
Wild animals	N/A
Field-collected samples	N/A
Ethics oversight	This research was fully approved by the Institutional Animal Care and Use Committee (IACUC) of both National University of Singapore and Ocean Park Hong Kong, with approval number 16-0319, and all experiments were performed in accordance with relevant guidelines and regulations

Note that full information on the approval of the study protocol must also be provided in the manuscript.
